# Author Correction: Therapeutic potential of KLF2-induced exosomal microRNAs in pulmonary hypertension

**DOI:** 10.1038/s41467-020-17273-7

**Published:** 2020-06-30

**Authors:** Hebah A. Sindi, Giusy Russomanno, Sandro Satta, Vahitha B. Abdul-Salam, Kyeong Beom Jo, Basma Qazi-Chaudhry, Alexander J. Ainscough, Robert Szulcek, Harm Jan Bogaard, Claire C. Morgan, Soni S. Pullamsetti, Mai M. Alzaydi, Christopher J. Rhodes, Roberto Piva, Christina A. Eichstaedt, Ekkehard Grünig, Martin R. Wilkins, Beata Wojciak-Stothard

**Affiliations:** 1 National Heart and Lung Institute, Imperial College London, London, UK; 2 University of Jeddah, College of Science, Department of Biology, Jeddah, Saudi Arabia; 3 Department of Physics, King’s College London UK, London, UK; 4 Amsterdam UMC, VU University Medical Center, Department of Pulmonary Diseases, Amsterdam Cardiovascular Sciences (ACS), Amsterdam, The Netherlands; 5 Max Planck Institute for Heart and Lung Research, Department of Lung Development and Remodeling, Member of the German Center for Lung Research (DZL), Bad Nauheim, Germany; 6 Department of Internal MedicineUniversities of Giessen and Marburg Lung Center (UGMLC), Member of the DZL, Justus Liebig University, Giessen, Germany; 7 National Center for Biotechnology, King Abdulaziz City for Science and Technology (KACST), Riyadh, Saudi Arabia; 8 Molecular Biotechnology Center, Department of Molecular Biotechnology and Health Sciences, University of Turin, Turin, Italy; 9 Centre for Pulmonary Hypertension, Thoraxclinic, Institute for Human Genetics, University of Heidelberg, Translational Lung Research Center (TLRC), German Center for Lung Research (DZL), Heidelberg, Germany; 10 Laboratory of Molecular Genetic Diagnostics, Institute of Human Genetics, Heidelberg University, Heidelberg, Germany

**Keywords:** Cell biology, Cardiovascular diseases

Correction to: *Nature Communications* 10.1038/s41467-020-14966-x, published online 4 March 2020.

The original version of this Article contained an error in the author affiliations.

Affiliation 2 incorrectly read ‘King Abdulaziz University, University of Jeddah, Jeddah, Saudi Arabia’ instead of the correct ‘University of Jeddah, College of Science, Department of Biology, Jeddah, Saudi Arabia’.

In addition the original version of this Article did not acknowledge Hebah Sindi as a corresponding author. This has now been corrected in both the PDF and HTML versions of the Article.

These errors have now been corrected in both the PDF and HTML versions of the Article.

