# Supplementary material for: Therapeutic potential of KLF2-induced exosomal microRNAs in pulmonary hypertension
Source: Nat Commun. 2020 Mar 4;11:1185. doi: 10.1038/s41467-020-14966-x (PMC7055281; doi:10.1038/s41467-020-14966-x)
Supplement: Supplementary file 1 — Supplementary Information [file 41467_2020_14966_MOESM1_ESM.pdf]

SUPPLEMENTARY INFORMATION

‘Therapeutic potential of KLF2 induced exosomal microRNAs in pulmonary hypertension’

Sindi H. A. et al.

SUPPLEMENTARY TABLES

Supplementary Table 1. List of differentially expressed KLF2-induced miRNAs; comparison between AdKLF2 and Adcontrol.

Assay	Fold change KLF/ Adcontrol	t-test p-value	Benjamini-Hochberg FDR
miR-19a-3p	-678.048044	1.20E-05	0.000944072
miR-216a-5p	-6.249853233	1.43E-05	0.000944072
miR-222-3p	-4.31940014	1.65E-05	0.000944072
miR-32-5p	-225.0788287	1.84E-05	0.000944072
miR-133b	-40.06999277	2.93E-05	0.001148513
miR-30c-5p	12.01858916	3.51E-05	0.001148513
miR-374a-5p	6.28982903	3.92E-05	0.001148513
miR-138-5p	-12.79690083	6.93E-05	0.001776134
miR-21-3p	-7.973287944	8.76E-05	0.001996236
let-7d-5p	13.07984808	0.000127348	0.002368834
miR-449a	-27.77133754	0.000142987	0.002368834
let-7a-5p	19.64292753	0.000155388	0.002368834
miR-125a-5p	2.945137006	0.000176063	0.002368834
miR-122-5p	-6.877036412	0.000176804	0.002368834
miR-34a-3p	-4.672742394	0.000183009	0.002368834
miR-887-3p	-3.359354582	0.000184885	0.002368834
miR-26a-5p	8.013287559	0.000200452	0.002417219
miR-18a-3p	5.309469128	0.000288552	0.003286283
miR-423-3p	4.024750615	0.000374417	0.003849113
miR-339-5p	1.944410728	0.000375523	0.003849113
miR-19b-3p	-251.2646609	0.000539535	0.00526689
miR-191-5p	12.33029968	0.00062517	0.005579615
miR-345-5p	-4.201525036	0.000626006	0.005579615
let-7d-3p	-6.094145735	0.000671162	0.005732841
miR-29b-3p	-52.17155579	0.000828729	0.006795578
miR-33a-5p	-206.9169658	0.000899755	0.006980693
miR-30a-5p	-2.962199179	0.000919408	0.006980693

let-7f-5p	18.76872495	0.000985341	0.007214106
miR-29c-3p	-3.467378229	0.001203602	0.008508224
miR-497-5p	-8.799499639	0.001339269	0.008636102
miR-374b-5p	10.96461448	0.00135387	0.008636102
let-7e-5p	10.39432937	0.001382485	0.008636102
miR-663a	18.88776335	0.001408569	0.008636102
miR-421	12.41733139	0.001471309	0.008636102
miR-106b-5p	-8.982369587	0.001474456	0.008636102
miR-324-5p	2.683113069	0.001674373	0.009534624
miR-155-5p	8.026920297	0.002256686	0.012319582
miR-30d-3p	1.74756363	0.002294868	0.012319582
miR-130b-3p	-1.986369701	0.002343725	0.012319582
miR-130a-3p	-8.283820051	0.002458013	0.012597316
miR-137	5.858978865	0.002562774	0.012813869
miR-744-5p	5.71063063	0.002687812	0.013119084
miR-652-3p	-2.16548831	0.002813207	0.013411803
miR-93-5p	2.384318594	0.003038364	0.014156015
miR-133a-3p	-46.92609151	0.003383325	0.015412926
let-7g-5p	4.132315381	0.003516855	0.01567294
miR-221-3p	5.24176575	0.003733719	0.01605327
miR-22-5p	-2.394003792	0.003758814	0.01605327
miR-181a-5p	2.343276512	0.004016857	0.016424314
miR-10a-5p	2.109710608	0.004048085	0.016424314
miR-34a-5p	-2.8914282	0.004086049	0.016424314
miR-136-5p	-13.53579255	0.004376448	0.017253306
miR-99b-5p	4.518692412	0.004597793	0.017783915
miR-100-5p	2.665107751	0.004983394	0.018918442
miR-30e-5p	-19.45237613	0.005084273	0.018950471
miR-382-5p	10.93265041	0.005274828	0.019309636
miR-193a-5p	1.934859045	0.005609297	0.020173787
miR-125b-5p	1.812592674	0.005772119	0.020218873
miR-101-3p	-49.83025244	0.00581909	0.020218873

miR-766-3p	5.455884548	0.005999583	0.020498576
miR-28-5p	7.653549225	0.006386619	0.021368795
miR-99a-5p	2.454410068	0.006481543	0.021368795
miR-423-5p	-2.015589866	0.006566996	0.021368795
miR-107	1.358338057	0.007072717	0.022654797
miR-146a-5p	-12.22799824	0.00729646	0.023011913
miR-26b-5p	2.897060378	0.007656657	0.023782039
miR-491-5p	2.466439294	0.00826466	0.025287392
miR-31-3p	-4.485743966	0.008458387	0.025499549
miR-326	1.420852038	0.009095812	0.027023791
miR-140-3p	-1.893160051	0.009455821	0.027542734
miR-151a-5p	4.104591929	0.009666552	0.027542734
let-7c-5p	7.258909766	0.009673545	0.027542734
miR-451a	-33.88772576	0.010688589	0.030015899
miR-663b	6.346162144	0.010949352	0.030332664
miR-409-3p	11.84976264	0.011926117	0.032598052
miR-181b-5p	4.326400767	0.012744974	0.034284009
miR-181c-5p	-3.344480262	0.01302467	0.034284009
miR-15a-5p	-6.149100492	0.013044647	0.034284009
miR-548b-3p	-13.27156988	0.013937329	0.036166487
miR-181d-5p	2.469456985	0.014446816	0.037019965
miR-425-5p	1.690974342	0.014929626	0.037784856
miR-103a-3p	2.128636617	0.019031225	0.046863267
miR-22-3p	-20.27394674	0.019034339	0.046863267
miR-877-5p	2.149441078	0.01920251	0.046863267
miR-29a-3p	-1.626595312	0.020069792	0.048403616
miR-30e-3p	2.358635645	0.020411747	0.048655908

Supplementary Table 2. Eight selected miRNAs upregulated by KLF2 and reduced in human PAH or hypoxic and MCT rat models of PAH.

	**miRNA ID in Ingenuity Pathway Analysis program (IPA)**
1	let-7a-5p (and other miRNAs w/seed GAGGUAG)
2	miR-10a-5p (and other miRNAs w/seed ACCCUGU)
3	miR-125b-5p (and other miRNAs w/seed CCCUGAG)
4	miR-181a-5p (and other miRNAs w/seed ACAUUCA)
5	miR-191-5p (and other miRNAs w/seed AACGGAA)
6	miR-30a-3p (and other miRNAs w/seed UUUCAGU)
7	miR-30c-5p (and other miRNAs w/seed GUAAACA)
8	miR-324-5p (and other miRNAs w/seed GCAUCCC)

Supplementary Table 3. Predicted and RNASeq-validated targets down-regulated by miR-181 and miR-324 associated with pathways regulating cell proliferation, inflammation and apoptosis.

NO	miR-181a-5p targets (downregulated)	Name/Function
1.	**ACTA2**	Actin, alpha 2, smooth muscle, aorta; cell motility, proliferation
2.	**AP1S3**	Subunit of clathrin-associated adaptor protein complex 1; plays a role in protein sorting in the late-Golgi/trans-Golgi network (TGN) and/or endosomes. Involved in TLR3 trafficking
3.	**C15orf48**	Chromosome 15 open reading frame 48
4.	**CARD11**	Caspase recruitment domain family 11; T-cell activation, NF-kappa-B activation. Stimulates the phosphorylation of BCL10
5.	**CBX4**	Chromobox homolog 4; SUMO-protein ligase which facilitates SUMO1 conjugation by UBE2I. Involved in the sumoylation, regulates p53/TP53 transcriptional activation resulting in p21/CDKN1A expression.
6.	**CCL8**	CCL8 - Chemokine (C-C motif) ligand 8; Chemotactic factor that attracts monocytes, lymphocytes, basophils and eosinophils.
7.	**CD69**	Early activation antigen CD69; Involved in lymphocyte proliferation and functions as a signal transmitting receptor in lymphocytes, natural killer (NK) cells, and platelets
8.	**CFAP161**	Chromosome 15 open reading frame 26; May play a role in motile cilia function, possibly by acting on dynein arm assembly
9.	**CXCL1**	Chemokine (C-X-C motif) ligand 1; chemotactic for neutrophils. May play a role in inflammation and exerts its effects on endothelial cells in an autocrine fashion.
10.	**CXCL9**	Chemokine (C-X-C motif) ligand 9; affects the growth, movement, or activation state of cells that participate in immune and inflammatory response. Chemotactic for activated T-cells.
11.	**DUSP10**	Dual specificity phosphatase 10; Protein phosphatase involved in the inactivation of MAP kinases. Has a specificity for the MAPK11/MAPK12/MAPK13/MAPK14 subfamily.
12.	**FBN2**	Fibrillin 2; extracellular matrix component, occurs in association with elastin. Participates in elastic fiber assembly. Controls TGF-beta bioavailability and calibrates TGF-beta and BMP levels.

13.	**FHDC1**	FH2 domain containing 1
14.	**HMGB2**	High mobility group box 2; Multifunctional protein, may act in a redox sensitive manner. In the nucleus is involved in transcription, chromatin remodeling and V(D)J recombination. Acts to promote activities on various gene promoters by enhancing transcription factor binding
15.	**HOXB8**	Homeobox B8; Sequence-specific transcription factor which is part of a developmental regulatory system that provides cells with specific positional identities on the anterior-posterior axis
16.	**IL1A**	Interleukin 1, alpha; stimulates thymocyte proliferation, B-cell maturation and proliferation, and fibroblast growth factor activity. Involved in the inflammatory response, reported to stimulate the release of prostaglandin and collagenase
17.	**LIF**	Leukemia inhibitory factor; induction of hematopoietic differentiation in normal and myeloid leukemia cells, the induction of neuronal cell differentiation, and the stimulation of acute-phase protein synthesis in hepatocytes
18.	**LRRC32**	Leucine rich repeat containing 32
19.	**MAMDC2**	MAM domain containing 2
20.	**MATN3**	Matrilin 3; Major component of the extracellular matrix of cartilage and may play a role in the formation of extracellular filamentous networks
21.	**MMP10**	Matrix metallopeptidase 10 (stromelysin 2); Can degrade fibronectin, gelatins of type I, III, IV, and V; weakly collagens III, IV, and V. Activates procollagenase
22.	**NOTCH4**	Notch 4; Functions as a receptor for membrane-bound ligands Jagged1, Jagged2 and Delta1 to regulate cell-fate determination. Affects the implementation of differentiation, proliferation and apoptotic programs. Regulates branching morphogenesis in the developing vascular system
23.	**NPIPA7**	NPIPA7 - Uncharacterized protein
24.	**NR4A3**	Nuclear receptor subfamily 4, group A, member 3; Transcriptional activator. Plays a role in the regulation of proliferation, survival and differentiation of many different cell types and also in metabolism and inflammation. Promotes mitogen-induced vascular smooth muscle cell proliferation

25.	**NSUN7**	NOP2/Sun domain family, member 7; May have S-adenosyl-L-methionine-dependent methyl- transferase activity
26.	**OGFRL1**	Opioid growth factor receptor-like 1
27.	**PRG2**	Proteoglycan 2, bone marrow (natural killer cell activator, eosinophil granule major basic protein); Cytotoxin and helminthotoxin. Also induces non-cytolytic histamine release from human basophils.
28.	**TNF**	Tumor necrosis factor; Cytokine that can induce cell death of certain tumor cell lines. Potent pro-inflammatory agent. It is potent pyrogen causing fever by direct action or by stimulation of interleukin-1 secretion and is implicated in the induction of cachexia, Under certain conditions it can stimulate cell proliferation and induce cell differentiation.
29.	**TTC39A**	Tetratricopeptide repeat domain 39A
30.	**UNC5A**	Unc-5 homolog A (C. elegans); Receptor for netrin required for axon guidance. It also acts as a dependence receptor required for apoptosis induction when not associated with netrin ligand
31.	**ZFP28**	Zinc finger protein 28 homolog (mouse); May be involved in transcriptional regulation. May have a role in embryonic development
32.	**ZNF256**	Zinc finger protein 256; Transcriptional repressor that plays a role in cell proliferation. Requires TRIM28 for its activity
33.	**ZNF558**	Zinc finger protein 558; May be involved in transcriptional regulation
34.	**ZNF563**	Zinc finger protein 563; May be involved in transcriptional regulation
35.	**ZNF582**	Zinc finger protein 582; May be involved in transcriptional regulation
36.	**ZNF788**	Zinc finger family member 788

**NO**	**miR-324-5p targets (downregulated)**	**Function**
1.	**CDK18**	Cyclin-dependent kinase 18, regulation of cell cycle
2.	**CD69**	Early activation antigen CD69; Involved in lymphocyte proliferation and functions as a signal transmitting receptor in lymphocytes, natural killer (NK) cells, and platelets
3.	**CLDN6**	Claudin 6; Plays a major role in tight junction-specific obliteration of the intercellular space

4.	**CORO2B**	Coronin, actin binding protein, 2B
5.	**CRABP2**	Cellular retinoic acid binding protein 2; Transports retinoic acid to the nucleus.
6.	**CYTH4**	Cytohesin 4; Promotes guanine-nucleotide exchange on ARF1 and ARF5. Promotes the activation of ARF factors through replacement of GDP with GTP
7.	**DCLK3**	Doublecortin-like kinase 3
8.	**ELFN1**	extracellular leucine-rich repeat and fibronectin type III domain containing 1; inhibits phosphatase activity of protein phosphatase 1 (PP1) complexes
9.	**ETS1**	V-ets erythroblastosis virus E26 oncogene homolog 1 (avian); Transcription factor. Directly controls the expression of cytokine and chemokine genes in a wide variety of different cellular contexts. Regulates cell differentiation, survival, proliferation, migration and angiogenesis.
10.	**F2RL3**	coagulation factor II (thrombin) receptor-like 3; Receptor for activated thrombin; plays a role in platelets activation
11.	**FAXDC2**	Chromosome 5 open reading frame 4
12.	**GFPT2**	Glutamine-fructose-6-phosphate transaminase 2; Controls the flux of glucose into the hexosamine pathway. Involved in regulating the availability of precursors for N- and O-linked glycosylation
13.	**GGT7**	Gamma-glutamyltransferase 7; Cleaves glutathione conjugates
14.	**GLI1**	GLI family zinc finger 1; Acts as a transcriptional activator. Regulator of cell proliferation and differentiation
15.	**GPR37L1**	G protein-coupled receptor 37 like 1; Regulates endocytosis and ERK phosphorylation cascade
16.	**GPX3**	Glutathione peroxidase 3 (plasma); Protects cells and enzymes from oxidative damage
17.	**GSG1L**	GSG1-like; As a component of the inner core of AMPAR complex
18.	**HIST2H4B**	Histone cluster 2, H4b
19.	**IRF8**	Interferon regulatory factor 8; Plays a negative regulatory role in cells of the immune system.
20.	**LDLRAD4**	Low density lipoprotein receptor class A domain containing 4; Regulator of TGF-beta signalling
21.	**MAPK13**	Mitogen-activated protein kinase 13; MAPK13 regulates cellular responses evoked by extracellular stimuli such as pro-inflammatory cytokines or physical stress
22.	**MICA**	MHC class I polypeptide-related sequence A
23.	**NFATC2**	Nuclear factor of activated T-cells, calcineurin-dependent 2; Plays a role in the inducible expression of IL-2, IL-3, IL-4, TNF-alpha or GM-CSF. Promotes invasive migration of cells
24.	**NKAIN1**	Na+/K+ transporting ATPase interacting 1

25.	**PRKCG**	Protein kinase C, gamma; Calcium-activated, phospholipid- and diacylglycerol (DAG)-dependent serine/threonine-protein kinase. Modulates cell survival after ischemia, and inhibition of gap junction activity after oxidative stress
26.	**RCAN1**	Regulator of calcineurin 1; Inhibits calcineurin-dependent transcriptional responses
27.	**RHCG**	Rh family, C glycoprotein; Functions as an electroneutral and bidirectional ammonium transporter. May regulate transepithelial ammonia secretion
28.	**SH2D5**	SH2 domain containing 5; May be involved in control of Rac-GTP levels
29.	**SH3RF2**	SH3 domain containing ring finger 2; Inhibits PPP1CA phosphatase activity. May be a E3 ubiquitin-protein ligase and play a role in cardiac function
30.	**SLC38A5**	Solute carrier family 38, member 5; Functions as a sodium-dependent amino acid transporter which countertransport protons
31.	**SLC6A4**	Solute carrier family 6 (neurotransmitter transporter, serotonin), member 4; Serotonin transporter, plays a key role in mediating regulation of the availability of serotonin
32.	**SOCS1**	Suppressor of cytokine signalling 1; Major regulator of signalling by interleukin 6 (IL6)
33.	**SPECC1**	Sperm antigen with calponin homology and coiled-coil domains 1
34.	**XG**	Glycoprotein, blood group antigen
35.	**TSPYL5**	TSPY-like 5; Involved in modulation of cell growth and cellular response stress probably via regulation of the Akt signalling pathway. Suppresses p53/TP53 protein levels and promotes its ubiquitination
36.	**ZFP3**	Zinc finger protein 3 homolog (mouse); May be involved in transcriptional regulation
37.	**ZNF773**	Zinc finger protein 773; May be involved in transcriptional regulation

Supplementary Table 4. Predicted and RNASeq-validated targets up-regulated by miR-181and miR-324

**NO**	**miR-181a-5p targets (upregulated)**	**Name/Function**
1.	**BAG2**	BAG family molecular chaperone regulator 2; Co-chaperone for HSP70 and HSC70 chaperone proteins.
2.	**CTGF**	Connective tissue growth factor; major mitoattractant secreted by vascular endothelial

		cells. Promotes proliferation and differentiation of chondrocytes. Mediates heparin- and divalent cation-dependent cell adhesion in many cell types including fibroblasts, myofibroblasts, endothelial and epithelial cells. Enhances fibroblast growth factor-induced DNA synthesis;
3.	**NEK7**	serine/threonine-protein kinase Nek7; Regulates mitotic cell cycle progression.
4.	**NOVA1**	NA-binding protein Nova-1; May regulate RNA splicing or metabolism
5.	**PI4K2B**	Phosphatidylinositol 4-kinase type 2-beta; Together with PI4K2A and the type III PI4Ks (PIK4CA and PIK4CB) it contributes to the overall PI4-kinase activity of the cell.
6.	**SIX2**	Homeobox protein SIX2; Transcription factor that plays an important role in the development of several organs, including kidney, skull and stomach
7.	**SMN1/SMN2**	Survival of motor neuron 1/2, telomeric; The SMN complex plays a catalyst role in the assembly of small nuclear ribonucleoproteins (snRNPs)
8.	**TSPAN8**	etraspanin-8; Tetraspanin 8; Belongs to the tetraspanin (TM4SF) family
**NO**	**miR-324-5p targets (upregulated)**	**Name/Function**
1.	**CLMP**	CXADR-like membrane protein; May be involved in the cell-cell adhesion.
2.	**GJC1**	Gap junction gamma-1 protein; regulates cell to cell communication
3.	**HRCT1**	Histidine rich carboxyl terminus 1
4.	**ITGBL1**	Integrin subunit beta like 1
5.	**LTBP2**	Latent-transforming growth factor beta-binding protein 2; May play an integral structural role in elastic-fiber architectural organization and/or assembly; Latent transforming growth factor beta binding proteins
6.	**MICA**	MHC class I polypeptide-related sequence A; Acts as a stress-induced self-antigen recognized by gamma delta T- cells.
7.	**MMRN1**	Multimerin-1; Carrier protein for platelet (but not plasma) factor V/Va.
8.	**PSME3**	Proteasome activator complex subunit 3; Subunit of the 11S REG-gamma proteasome regulator. May be involved in cell cycle regulation.

Supplementary Table 5. Kyoto Encyclopedia of Genes and Genomes (KEGG) pathway associations of miR-181 and miR-324 targets.

**Pathway ID**	**Pathway Description**	**Genes**	**FDR**
hsa04668	TNF signaling pathway	TNF, TRADD, IKBKG, RIPK1,GLI1, TAB2, MAPK13, LIF	2.41e-06
hsa04380	Osteoclast differentiation	TRADD, TAB2, IKBKG, MAPK13, NFATC2, SOCS1	5.74e-05
hsa04657	IL-17 signaling pathway	TNF, TAB2, TRADD, IKBKG,MAPK13, CXCL1	0.00011
hsa04064	NF-kappa B signaling pathway	TNF, TRADD, TAB2, CARD11, RIPK1, GLI1	0.00011
hsa04620	Toll-like receptor signaling pathway	MAPK13, TRADD, TAB2, RIPK1, GLI1, CXCL9	0.00012
hsa04010	MAPK signaling pathway	TNF, TAB2, TRADD, IKBKG,IL1A,DUSP10, MAPK13, PRKCG	0.00036
hsa04621	NOD-like receptor signaling pathway	TNF, IKBG, GLI1, MAPK13, RIPK1, TAB2, CXCL1	0.00080
hsa04060	Cytokine-cytokine receptor interaction	TNF, LIF, CXCL9, CXCL8, CXCL1, IL1A, TRADD	0.0059
hsa04071	Sphingolipid signaling pathway	TRADD, TNF, MAPK13, PRKCG	0.0104
hsa04370	VEGF signaling pathway	MAPK13, NFATC2, PRKCG, CXCL8	0.0138
hsa05418	Fluid shear stress and atherosclerosis	IL1A, TNF, IKBKG, MAPK13	0.0145
hsa04210	Apoptosis	GLI1, TRADD, TNF, IKBKG, TAB2	0.0145
hsa04218	Cellular senescence	ETS1, NFATC2, MAPK13, IL1A,	0.0192
hsa05323	Rheumatoid arthritis	TNF, CXCL1, IL1A	0.0248
hsa04670	Leukocyte transmigration	MAPK13, PRKCG, NOTCH4	0.0370
hsa04940	Type I diabetes mellitus	TNF, IL1A	0.0403

Supplementary Table 6. Demographics of blood donors, IPAH patients and healthy volunteers. Data represented as median (range). Control (n=14), IPAH (n=12)

		Control	IPAH
Male/Females		3 per 11	1 per 11
Age (years)		30.0 (23.0 - 45.0)	38.33 (27.0 - 67.0)
Time from diagnosis (months)		-	13.0 (0.1 - 60.0)
mPAP (mmHg)		-	65.0 (28.0 - 94.0)
Six minute walk distance (m)		-	396.0 (300.0 – 540.0)
WHO Functional Class	I	-	1
	II	-	3
	III	-	5
	IV	-	3
Treatment naïve		-	2
Warfarin		-	4
Calcium Antagonists		-	1
ER Antagonists		-	7
PDE5 Inhibitors		-	8
Prostanoids		-	4
Statins		-	0

mPAP, mean Pulmonary Arterial Pressure; ER, Estrogen-Receptor; PDE5, Phosphodiesterase type 5.

Supplementary Table 7. Demographics of lung tissue donors, PAH patients and control individuals. PAH lung tissue samples were from 5 patients with IPAH and 1 patient with HPAH, while control samples were from uninvolved regions of lungs from lung carcinoma patients. Data represented as median (range). Control and IPAH n=6 per group.

	Control	IPAH
Male/Females	3 per 6	2 per 6
Age (years)	51.0 (27.0 - 59.0)	45.33 (28.0 - 51.0)
Time from diagnosis (months)	-	42 (18-96)
mPAP (mmHg)	*	60.0 (52.0 - 70.0)
Treatment Naïve	-	5
Prostanoids	-	1

mPAP; mean Pulmonary Arterial Pressure, * no effect on PAP

Supplementary Table 8.

TaqMan® Gene Expression Assays

Gene	Human Probe	Mouse Probe
KLF2	Hs00360439_g1	Mm00500486_g1
KLF4	Hs00358836_m1	
NOTCH4	Hs00965895_g1	Mm00440525_m1
ETS1	Hs00428293_m1	Mm01175819_m1
GAPDH	Hs02786624_g1	Mm99999915_g1
TNF-α	Hs00174128_m1	
MAPK18	Hs00559623_m1	

TaqMan® miRNA Assays

miRNA	Assay ID
miR-181a-5p	000480
miR-324-5p	000539
miR-32-5p	002109
U6 snRNA	001973

SUPPLEMENTARY FIGURES AND FIGURE LEGENDS

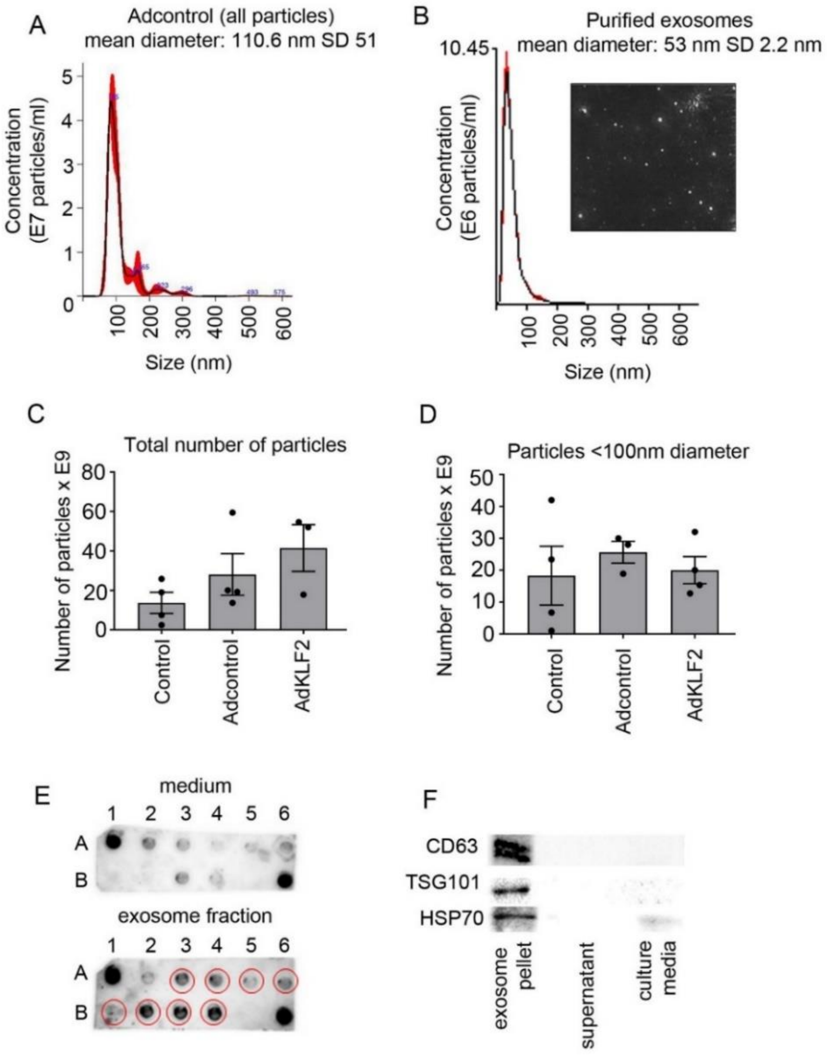

Supplementary Figure 1. Exosome quantification. (A) and (B) show particle distribution in conditioned media and purified exosome fraction from control HPAECs (Adcontrol), as indicated. (C) Total number of particles and (D) number of exosomes (particles <100 nm in diameter) in control (untreated) HPAECs and HPAECs infected with AdGFP (Adcontrol) or AdKLF2-GFP, as indicated. Quantification was carried out with NanoSight LM10 Particle Size Analyzer. In (C, D) bars are mean values ± s.e.m. n=4, no significant difference between groups; one-way ANOVA with Tukey post-test. (E) Representative images of exosomal marker microarray of conditioned medium and purified exosome fraction, as indicated. (F) Western blots show expression of exosomal markers CD63, TSG101 and HSP60 in exosome pellet, supernatant and culture media, as indicated (Exiqon exosome purification kit). All n numbers represent independent experiments. Source data are provided in Supplemental Source Data file.

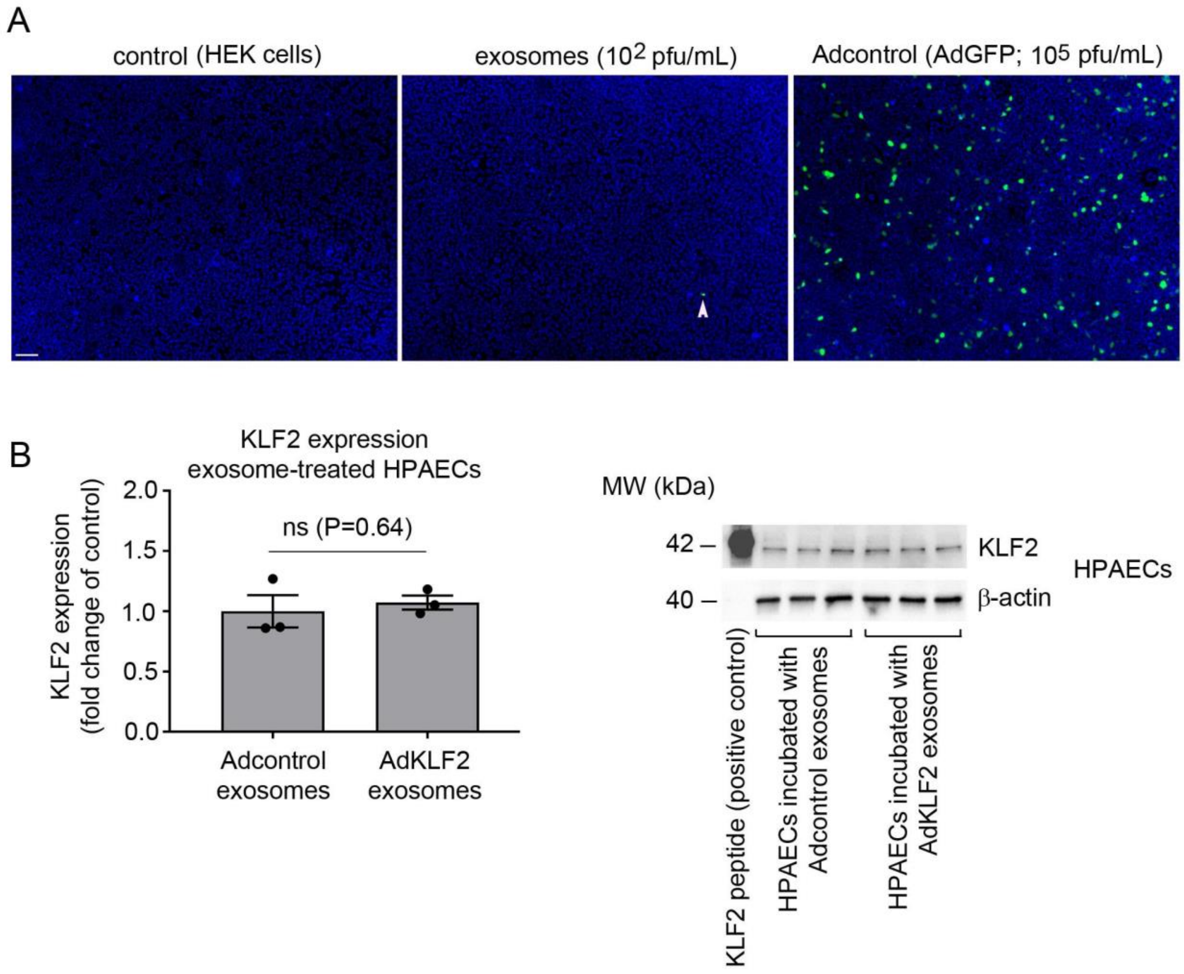

Supplementary Figure 2. Adenoviral particles in exosome fraction. (A) images of the untreated, exosome-treated (2-fold diluted exosome fraction) and adenovirus-infected HEK293 cells 48 hours post-treatment. Nuclei are blue (Hoechst), GFP-overexpressing cells are green and the arrowhead points to an infected (GFP-positive) cell. Bar=100µm. (B) KLF2 protein expression in HPAECs treated, as indicated. Values in (B) are mean fold-changes of untreated controls ± s.e.m., n=3. ns: non-significant, unpaired t-test. Corresponding western blot is shown on the right. Titration of the virus was carried out by preparing serial dilutions of the exosome fraction (10^{-1}-10^{-7}) in 96-well plates containing 1.5×10^{3} HEK293 cells per well. Plaque formation was scored following 10-day incubation. For experiments, the purified exosome fraction (containing 10^{2} pfu per mL) was further diluted 50-fold in culture media. All n numbers represent independent experiments. Source data are provided in Supplemental Source Data file.

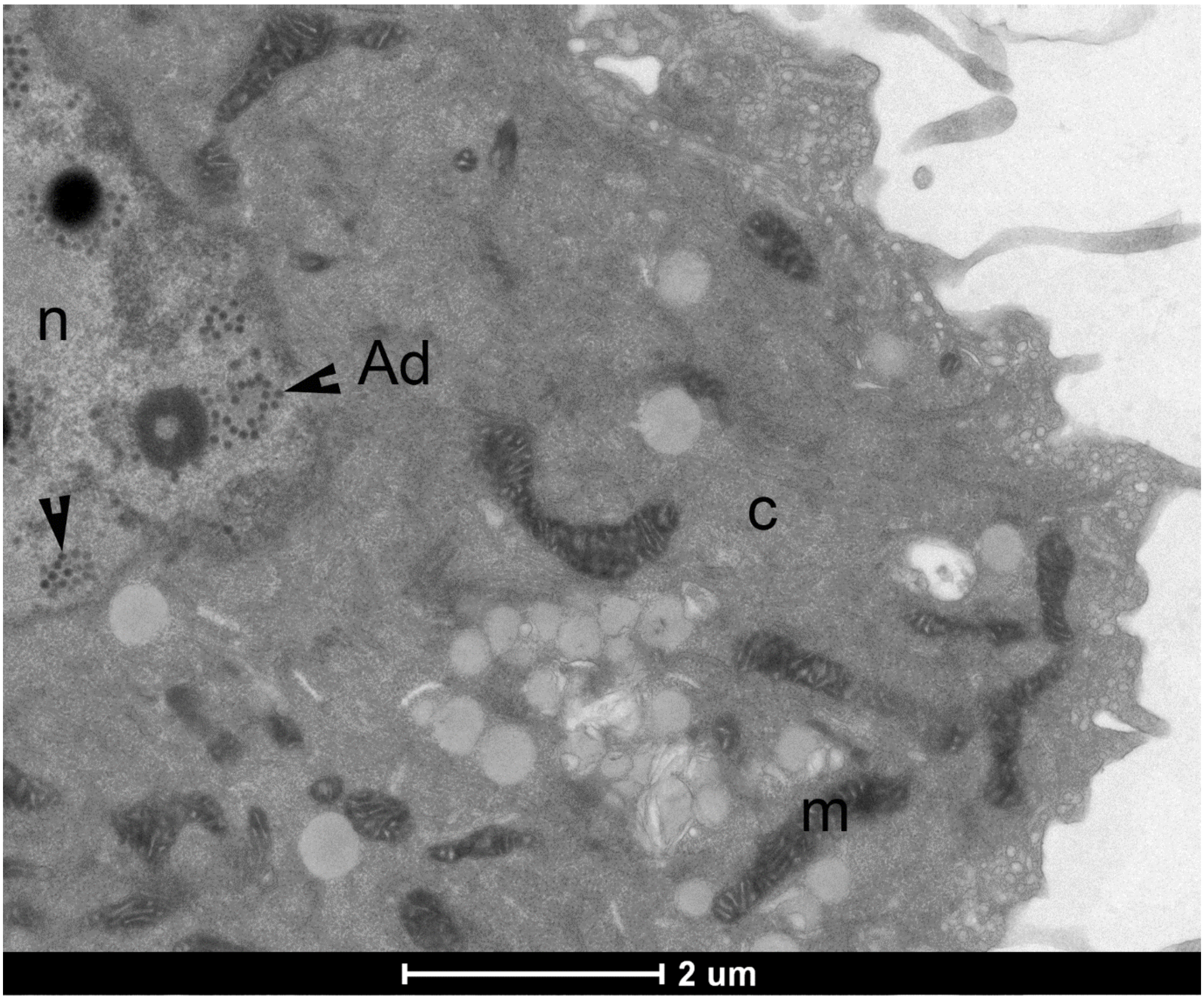

Supplementary Figure 3. Transmission electron microscopy of HPAEC infected with control adenoviruses 24 hours post-infection. C-cytoplasm; m-mitochondrion, n-nucleus, Ad-adenoviral particles. Bar=2µm.

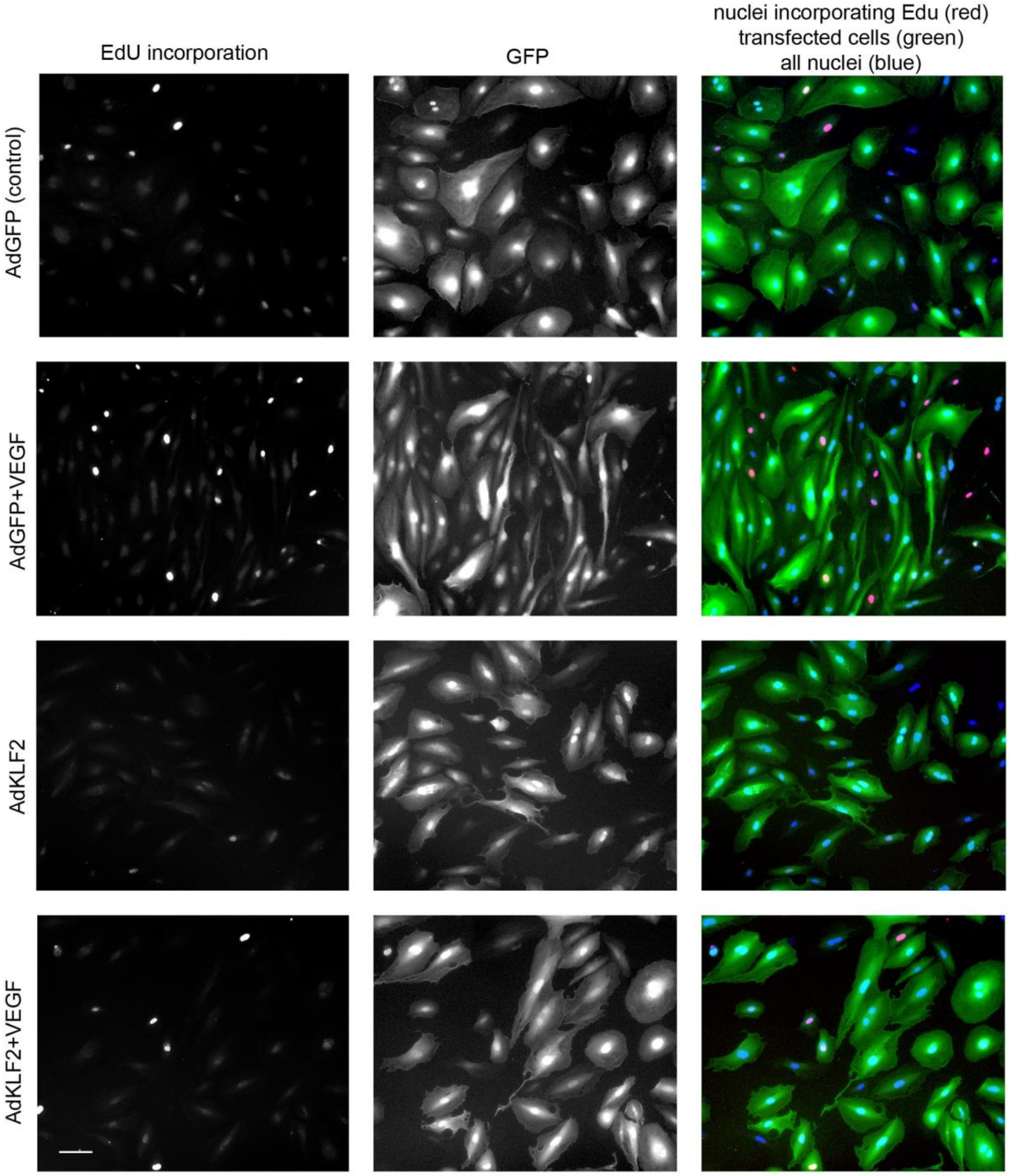

Supplementary Figure 4. Effect of KLF2 on endothelial cell proliferation. HPAECs overexpressing AdGFP or AdKLF2-GFP, pre-starved in growth factor-depleted media containing 2% serum, were stimulated with 50ng per mL VEGF for 18 hours. Proliferating cells were visualised in EdU incorporation assay. Bar=20μm.

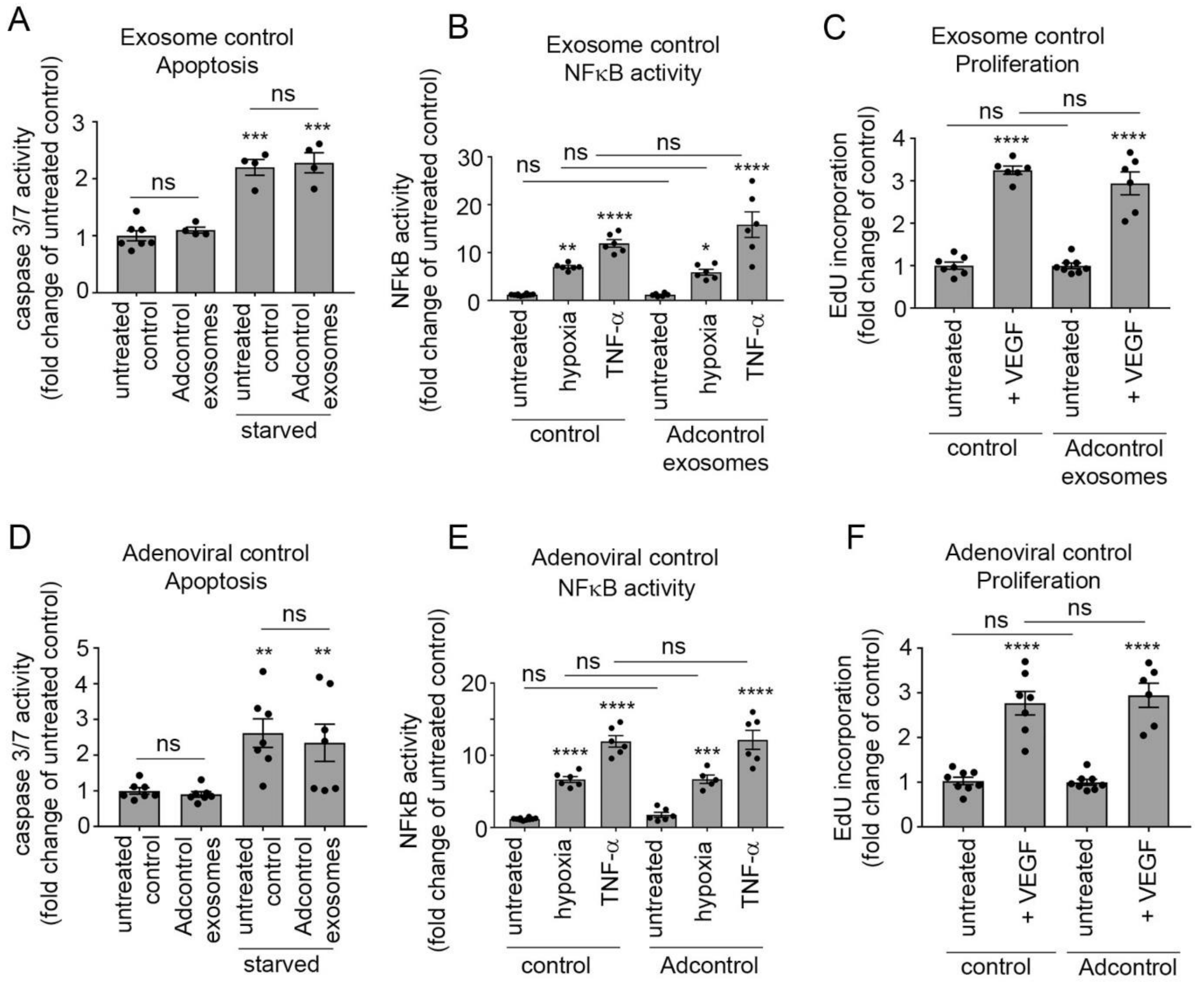

Supplementary Figure 5. **Effect of exosomal and adenoviral control treatement on endothelial cell apoptosis, inflammatory activation and proliferation.** Effects of (A, B, C) exosomes from HPAECs overexpressing GFP (Adcontrol exosomes) and (D, E, F) HPAECs infected with AdGFP (Adcontrol) on caspase 3/7 activation in serum-starved HPAECs (24h), hypoxia- and TNF-α)-induced (10 µg per L, 24h) NFκB activation and VEGF-induced (50ng per mL, 18h) cell proliferation in HPAECs, as indicated. *P<0.05; **P<0.01; ***P<0.001, ****P<0.0001 comparison with untreated controls; ns-non significant. One-way ANOVA with Tukey post-test. Values in (A-F) are mean fold-changes of untreated controls ± s.e.m., n=4-8. All n numbers represent independent experiments. Source data are provided in Supplemental Source Data file.

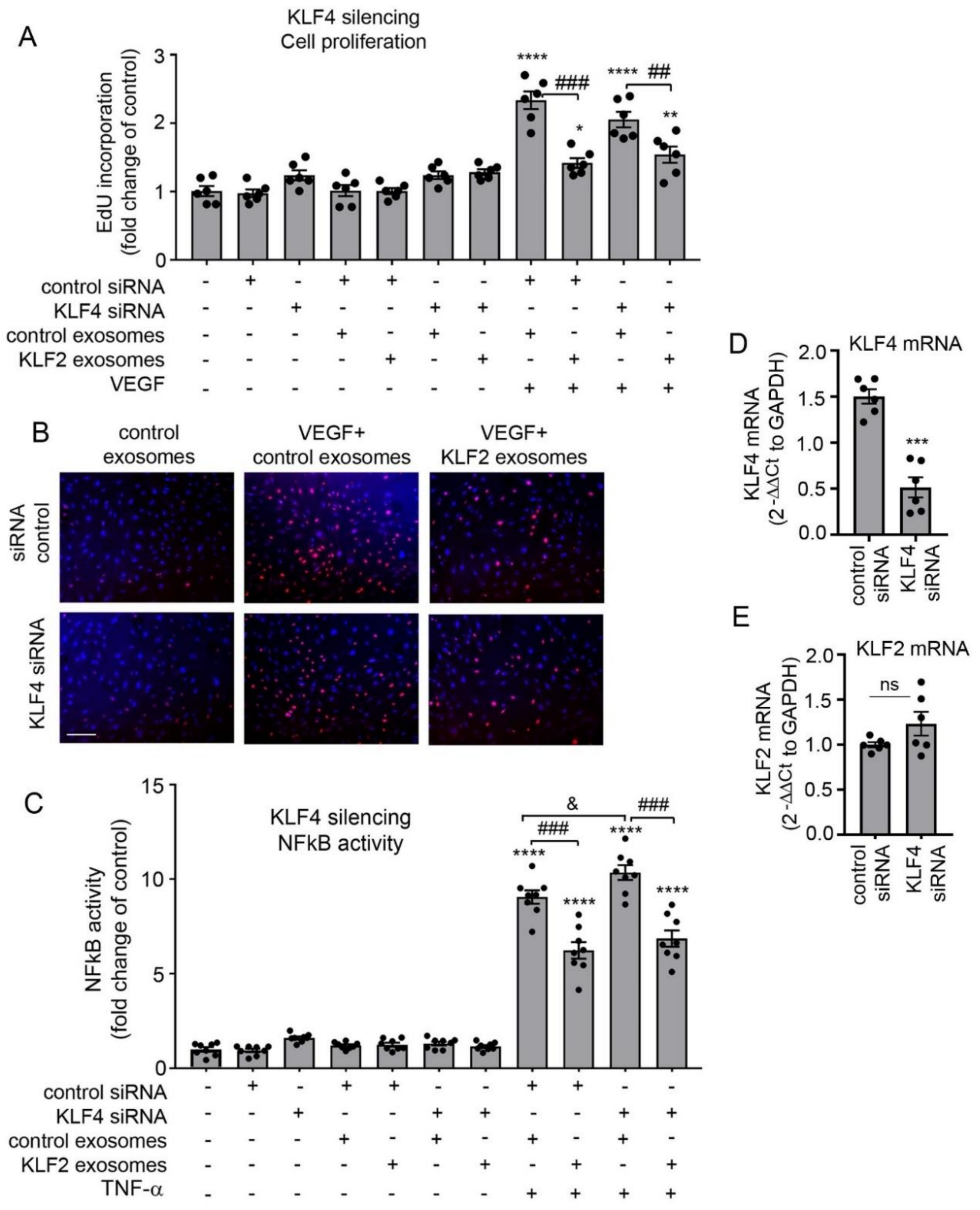

Supplementary Figure 6. Effects of KLF4 silencing on KLF2 exosome-induced responses in HPAECs. (A, B) VEGF-induced cell proliferation, (C) TNF-α-induced NFkB activity and (D, E) KLF4 and KLF2 mRNA levels in HPAECs transfected with control siRNA or KLF4 siRNA. (B) shows EdU incorporation in HPAECs treated with control siRNA or KLF4siRNA and stimulated with VEGF, with or without KLF2 exosomes, as indicated. All nuclei are blue, while EdU-incorporating nuclei are red. Bar=100µm.*P<0.05; **P<0.01; ***P<0.001; ****P<0.0001, comparisons with untreated control (A, C) or siRNA control (D, E). One-way ANOVA with Tukey post-test (A, C) or unpaired t-test (D, E), as appropriate. Values in (A, C) are mean fold-changes of untreated controls ± s.e.m., n=6-8; values in (D, E) are mean fold changes of control siRNA ± s.e.m., n=6; $^{\&}$P<0.05; $^{\#\#}$P<0.01; $^{\#\#\#}$P<0.001, comparisons, as indicated. ns – non significant. All n numbers represent independent experiments. Source data are provided in Supplemental Source Data file.

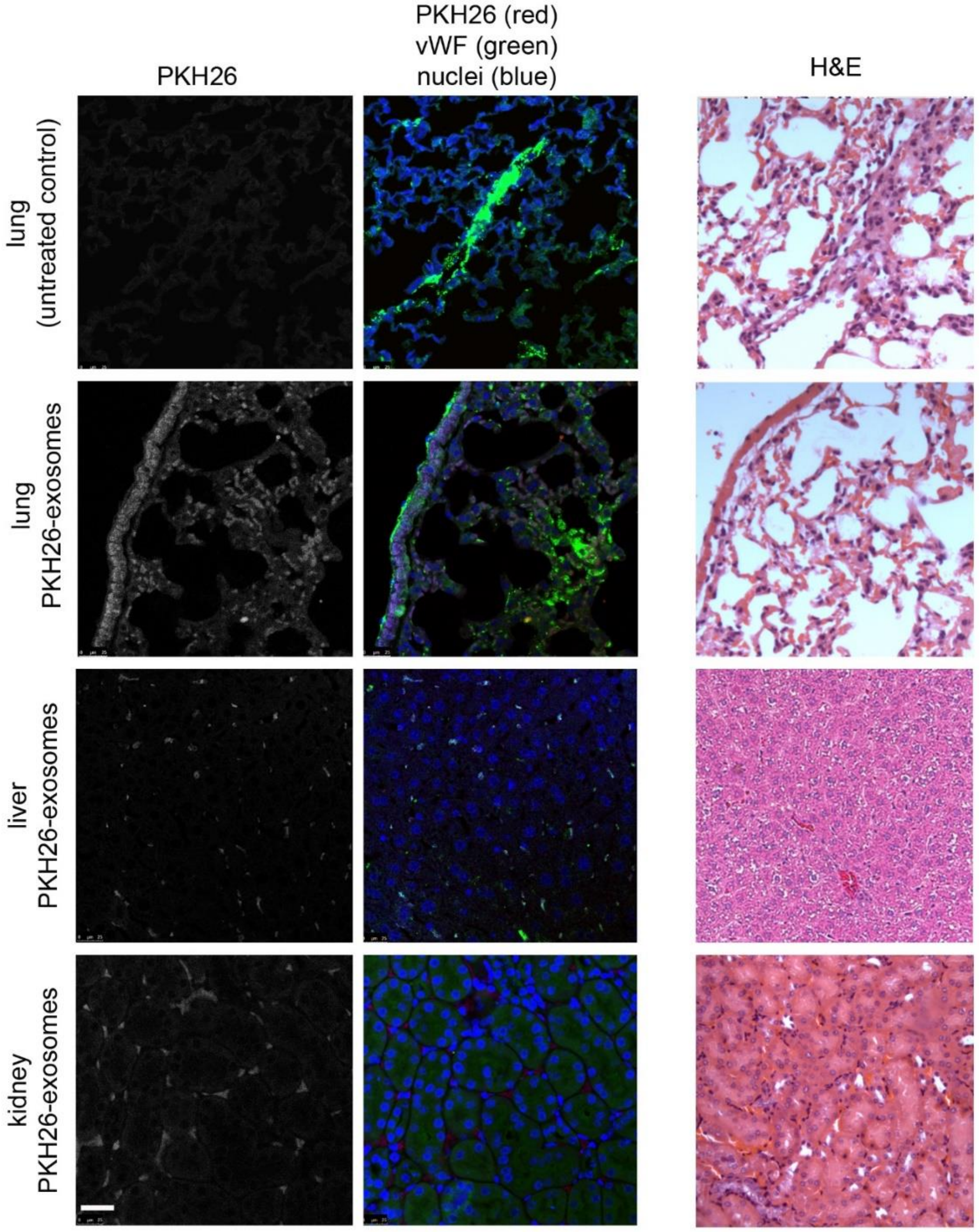

Supplementary Figure 7. Visualisation of PKF26-labelled exosomes in vivo. PKH26-labelled exosomes (~2x10^{11} particles in 100 µL of PBS) were injected into the tail veins of healthy mice. Distribution of exosomes in lung, liver and kidney was examined 4 hr post-injection. Endothelial localization was confirmed by co-immunostaining for vWF. Right panels shows H&E staining. Bar=25 µm.

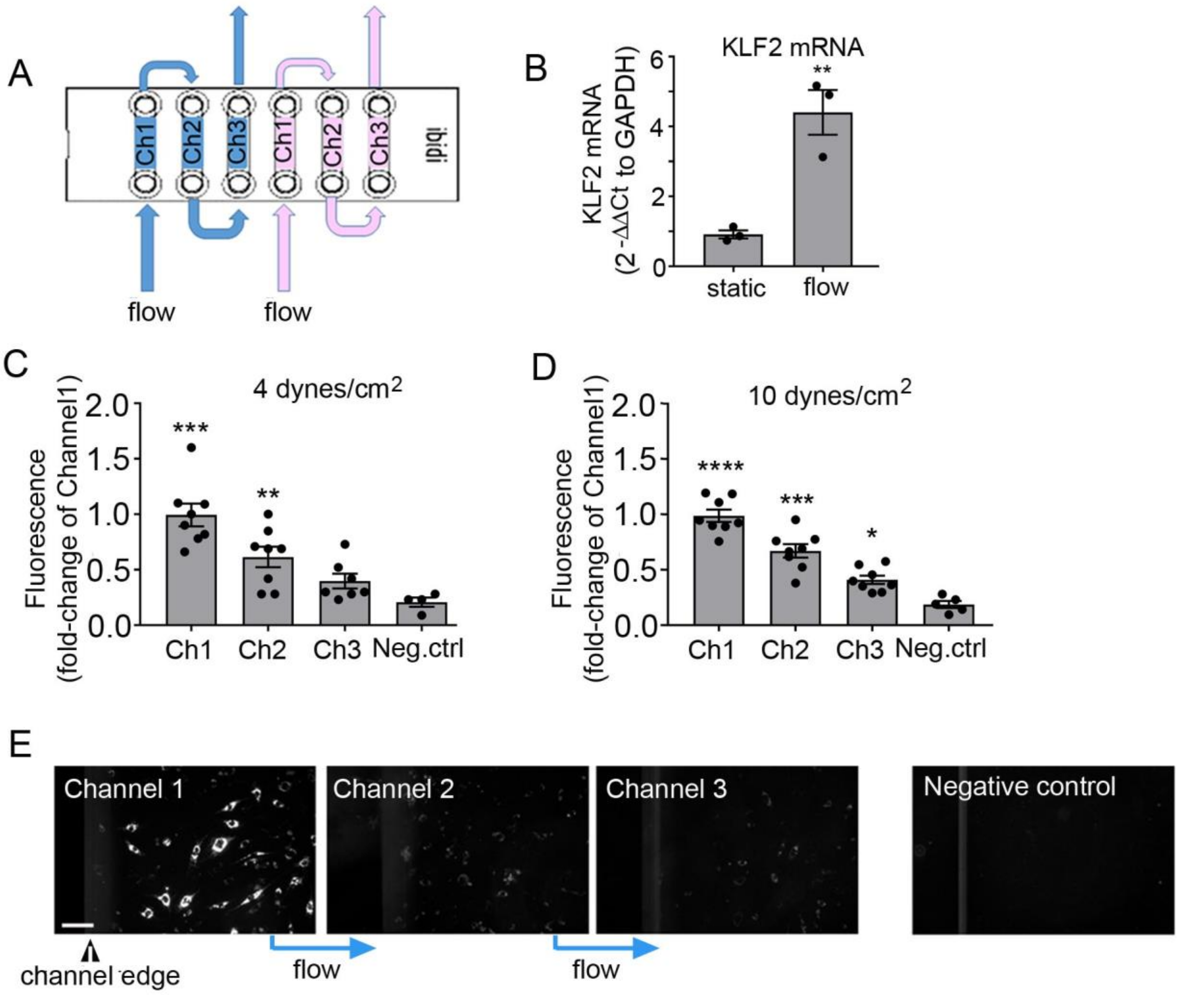

Supplementary Figure 8. miRNA transfer under flow. (A) Schematic representation of flow setup. (B) KLF2 mRNA in HPAECs stimulated by flow (C, D) Cy3-labelled miRNA transfer in HPAECs under flow (fold-change of fluorescence in Channel 1 ± s.e.m.). Cells in Channel 1 were transfected with Cy3-miR and washed several times to remove fluorescent miRNA. Flow of medium (4 dynes per cm^2, 24h) in (C) and (10 dynes per cm^2, 24h) in (D) was directed from Channel 1 to Channel 2 and Channel 3. (E) Representative images of cells in Channels 1, 2 and 3 under 4 dynes per cm^2 flow; Bar=50μm. *P<0.05, **P<0.01, ***P<0.001, ****P<0.0001, comparison with static or negative control (transfection reagent only). In (B) unpaired t-test n=3, in (C, D) one-way ANOVA with Tukey post-test; In (B) n=4 and in (C, D) n=4-8. All n numbers represent independent experiments. Source data are provided in Supplemental Source Data file.

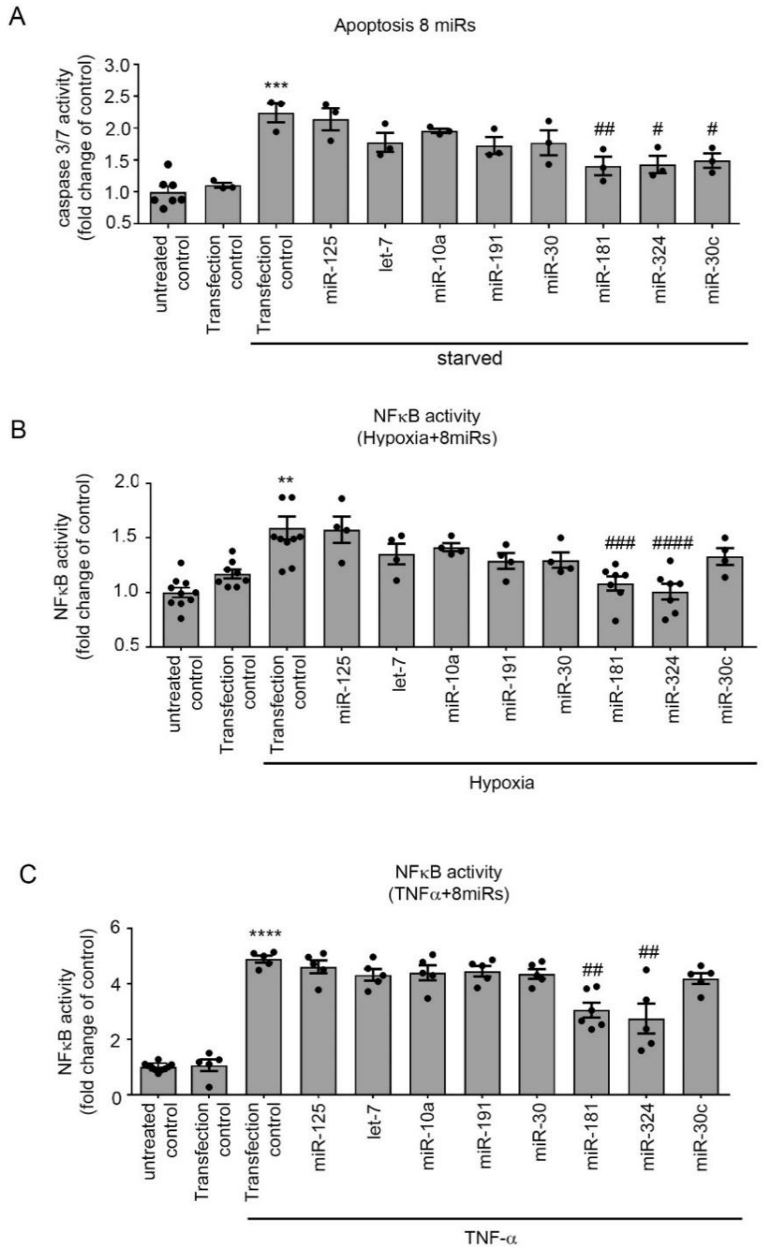

Supplementary Figure 9. Effects of 8 selected miRNAs on HPAEC function. (A) Apoptosis (caspase 3/7 activity) in serum-starved HPAECs transfected with control miRNA or miR-125, let-7, miR-10a, miR-191, miR-30, miR-181, miR-324, miR-30c, as indicated. (B, C) NFκB activity in HPAECs transfected with miRNAs and cultured in hypoxic conditions or treated with TNF-α (10 µg per L) for 24h, as indicated. Bars show mean fold-change of untreated, untransfected controls ± s.e.m. $^{**}P<0.01$, $^{***}P<0.001$, $^{****}P<0.0001$, comparison with transfection controls; $^{\#}P<0.05$; $^{\#\#}P<0.01$; $^{\#\#\#}P<0.001$; $^{\#\#\#\#}P<0.0001$, comparison with treatment controls. One-way ANOVA with Tukey post-test; n=3-8. All n numbers represent independent experiments. Source data are provided in Supplemental Source Data file.

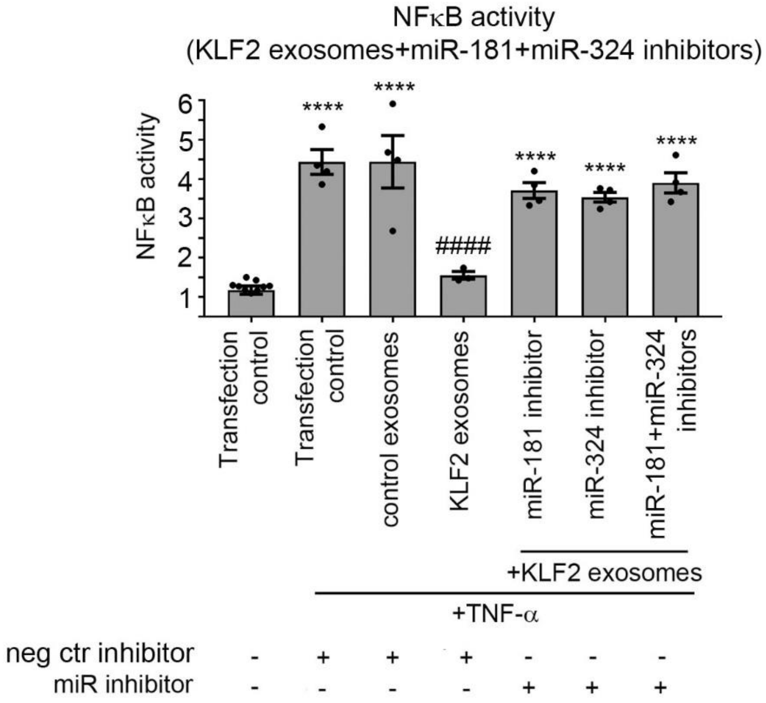

Supplementary Figure 10. miR-181 and miR-324 inhibitors reverse effects of KLF2 exosomes. AdNFκB-luc-infected HPAECs were treated with KLF2 exosomes isolated from endothelial cells transfected with control inhibitor or miR-181, miR-324 inhibitors, with or without TNF-α, as indicated. Bars show mean fold-change of luminescence in untreated, untransfected controls ± s.e.m. ****P<0.0001, comparison with untreated transfection controls; ####P<0.0001, comparison with treatment controls. One-way ANOVA with Tukey post-test, n=4-8. All n numbers represent independent experiments. Source data are provided in Supplemental Source Data file.

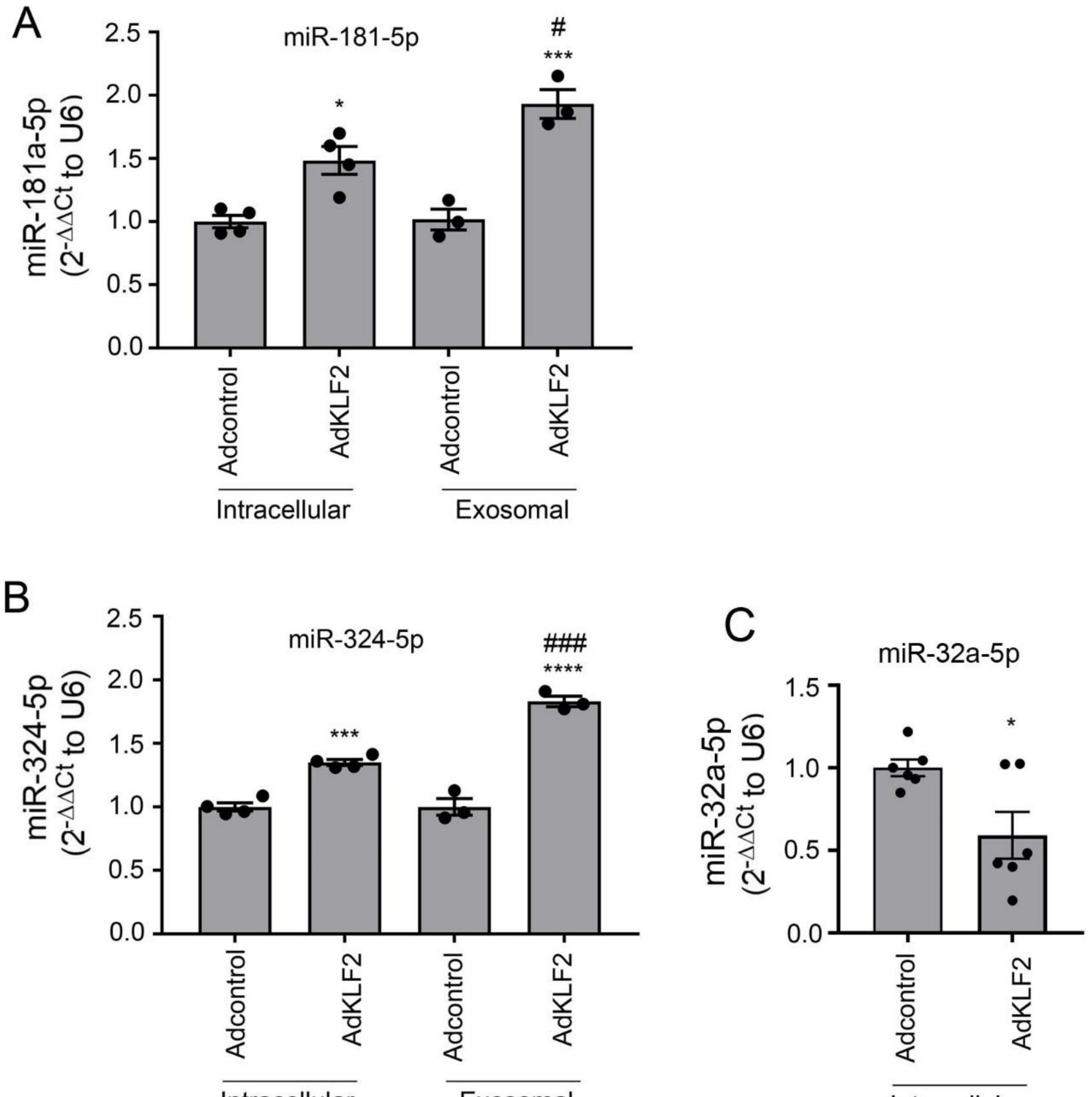

Supplementary Figure 11. KLF2 increases intracellular and exosomal levels of miR-181 and miR-324 in HPAECs. Intracellular and exosomal levels of (A) miR-181 and (B) miR-324 in HPAECs overexpressing AdGFP (Adcontrol) and AdKLF2 (24h). (C) Intracellular levels of miR-32a-5p are reduced in KLF2-overexpressing HPAECs, consistent with reduction of this miRNA in exosomes shown in Table S1. Bars show mean fold-change of Adcontrols ± s.e.m. *P<0.05, ***P<0.001, ****P<0.0001, comparison with intracellular or exosomal Adcontrols, as appropriate; $^{\#}$P<0.05, $^{\#\#\#}$P<0.001, comparison between intracellular and exosomal AdKLF2 levels; One-was ANOVA with Tukey post-test (A, B) or unpaired t-test (C). In (A, B) n=3-4, in (C) n=6. All n numbers represent independent experiments. Source data are provided in Supplemental Source Data file.

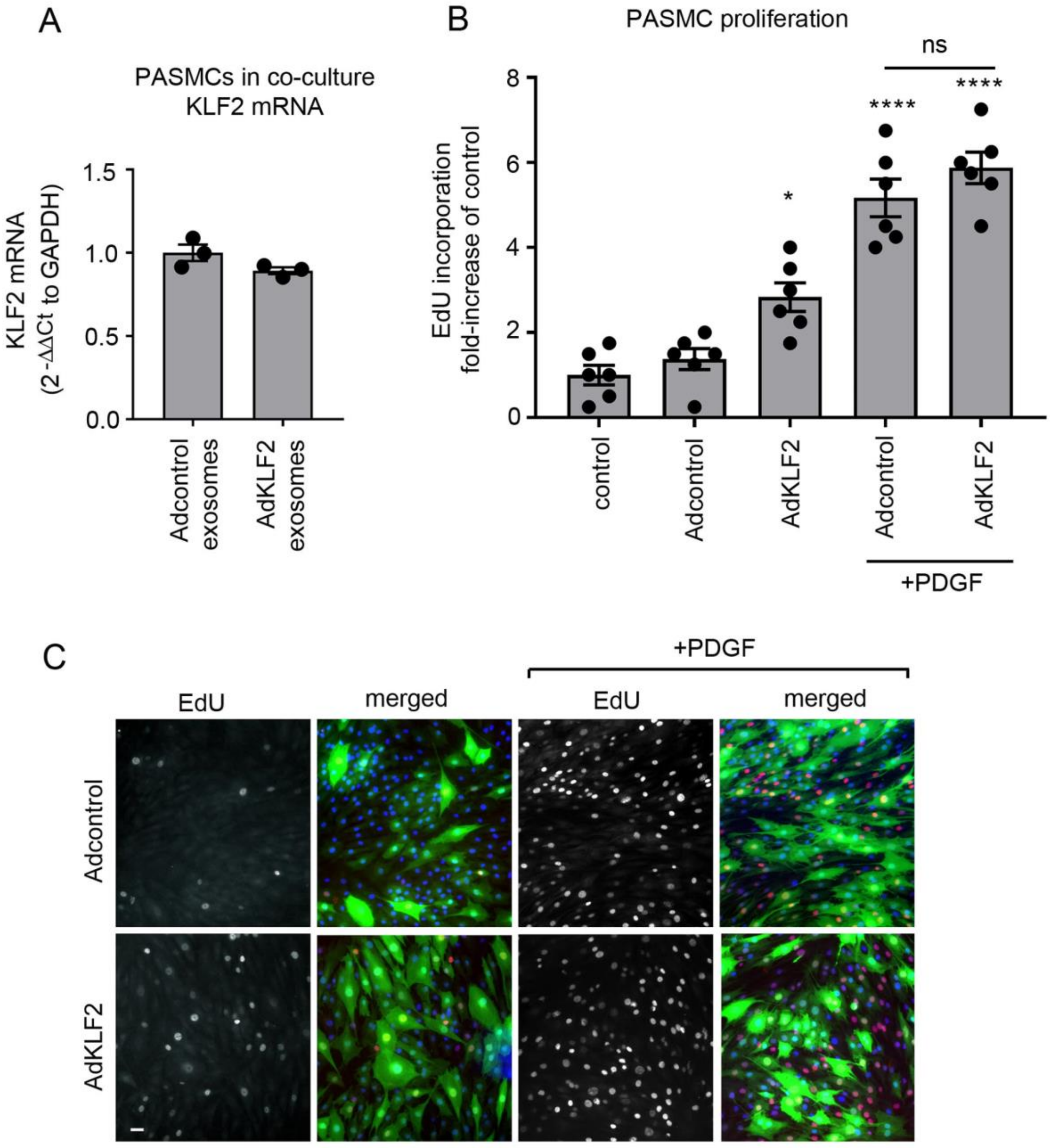

Supplementary Figure 12. Effects of KLF2 in HPASMCs. (A) KLF2 mRNA levels in HPASMCs treated with exosomes obtained from HPAECs treated with Adcontrol (Adcontrol exosomes) or AdKLF2 (AdKLF2 exosomes). (B, C) Graph and corresponding representative images showing effect of adenoviral overexpression of KLF2 in HPASMCs on PDGF-induced proliferation. In (C) all nuclei are blue (Hoechst), nuclei incorporating EdU are red, while cells infected with AdGFP (Adcontrol) or AdKLF2-GFP, are green. Bar=10µm. Values in (A, B) are mean fold-changes of Adcontrol or untreated controls ± s.e.m., n=3-6. *P<0.05; ****P<0.0001, comparison with the untreated control; unpaired t-test in (A) and one-way ANOVA with Tukey post-test in (B). All n numbers represent independent experiments. Source data are provided in Supplemental Source Data file.

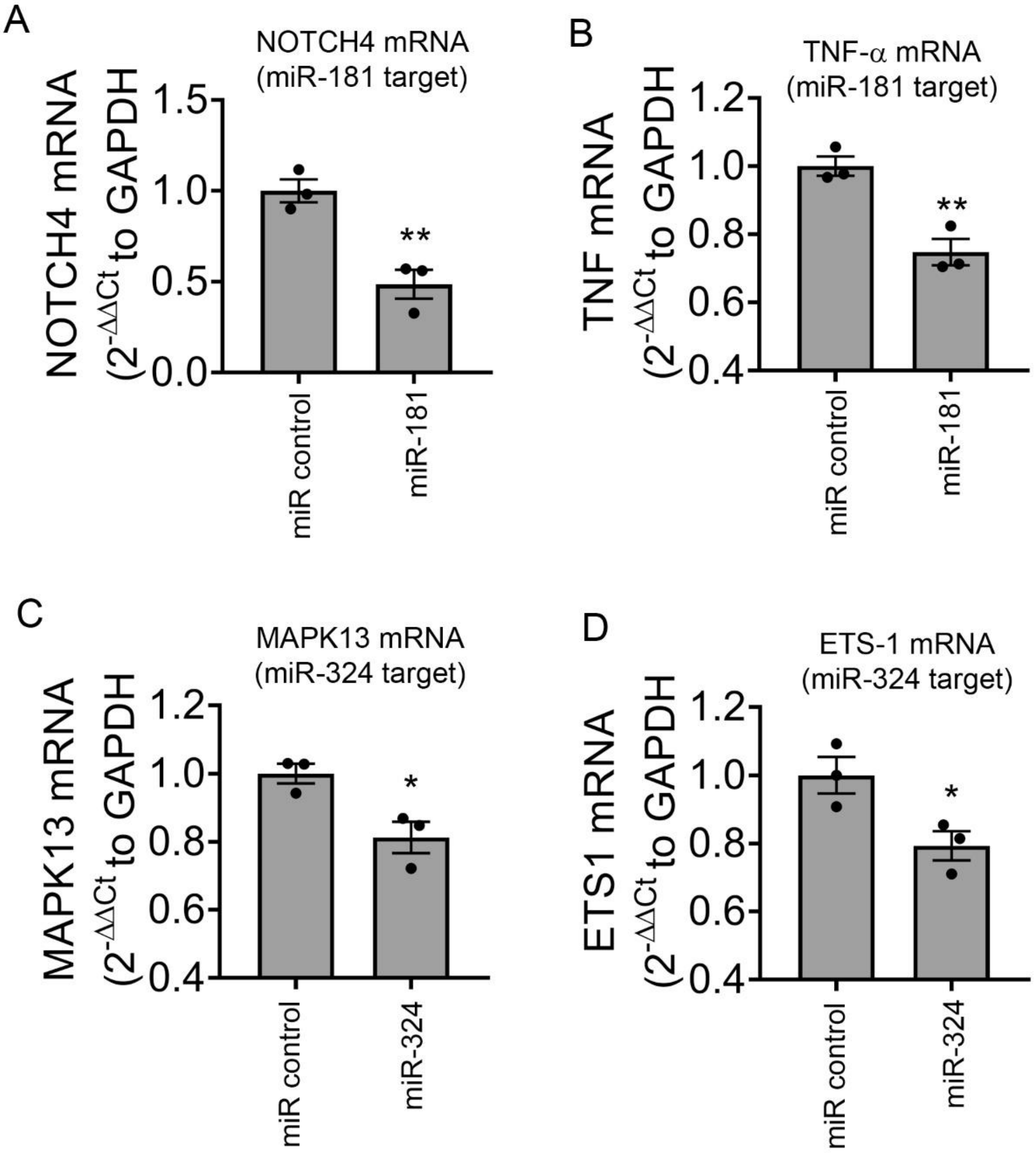

Supplementary Figure 13. Validation of changes in expression of selected miR-181 and miR-324 targets by qPCR 24 hours post-transfection. (A) Notch4 mRNA, (B) TNF-α mRNA, (C) MAPK13mRNA and (D) ETS-1 mRNA. *P<0.05; **P<0.01, comparison with miR control. Values in (A-D) are mean fold-changes of miR controls ± s.e.m., n=3; unpaired t-test. All n numbers represent independent experiments. Source data are provided in Supplemental Source Data file.

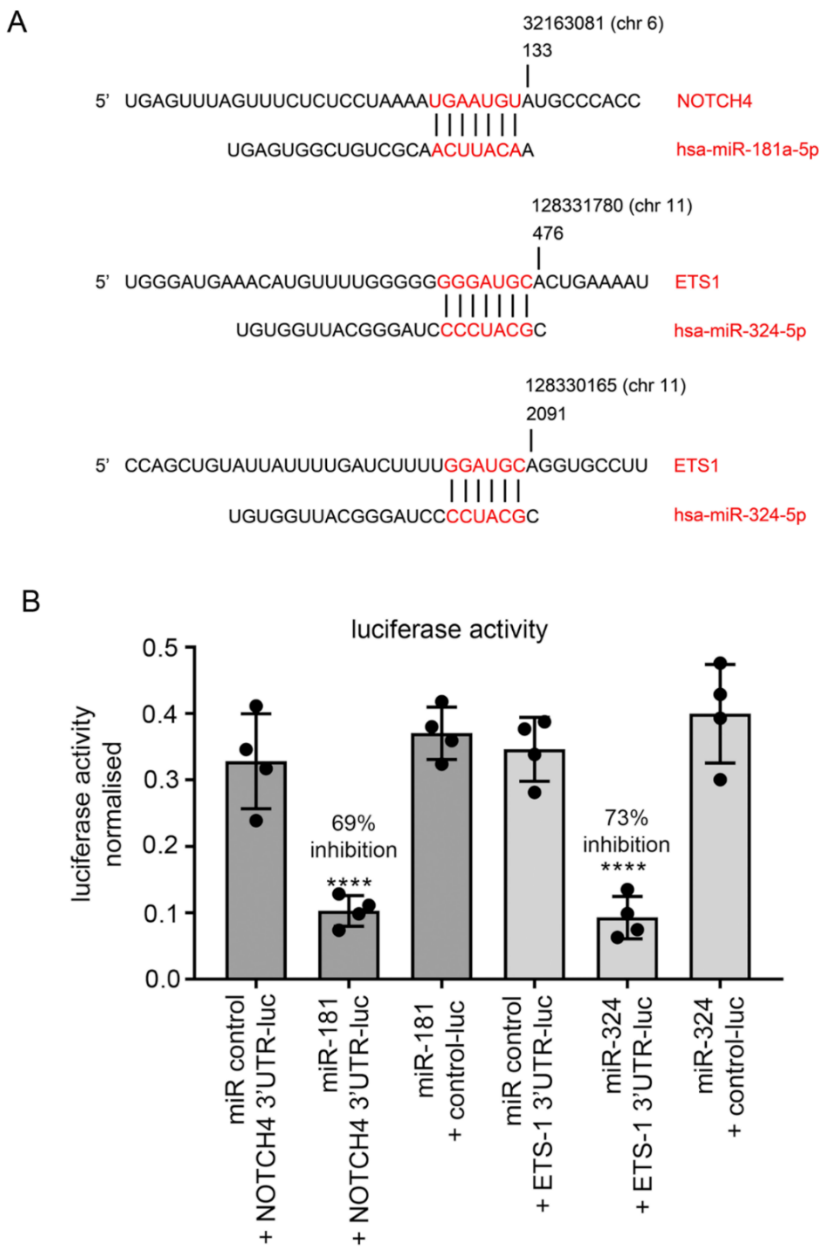

Supplementary Figure 14. miRNA-mediated targeting of Notch4 and ETS-1. (A) miR-181-5p and miR-324-5p binding sites of in Notch 4 or ETS-1 3'UTR (mirmap and microrna.org). (B) Validation of Notch4 and ETS-1 3'UTR targeting by miR-181 and miR-324; luciferase reporter assay. Cells were transfected with miR-181 or miR-324 and Notch4 or ETS-1 luciferase 3'UTR reporter constructs or plasmid control (control-luc), as appropriate. (B) shows mean values of Firefly luminescence normalised to Renilla luminescence ± s.e.m. ****P<0.0001, comparison with respective miR controls; unpaired t-test, n=4. All n numbers represent independent experiments. Source data are provided in Supplemental Source Data file.

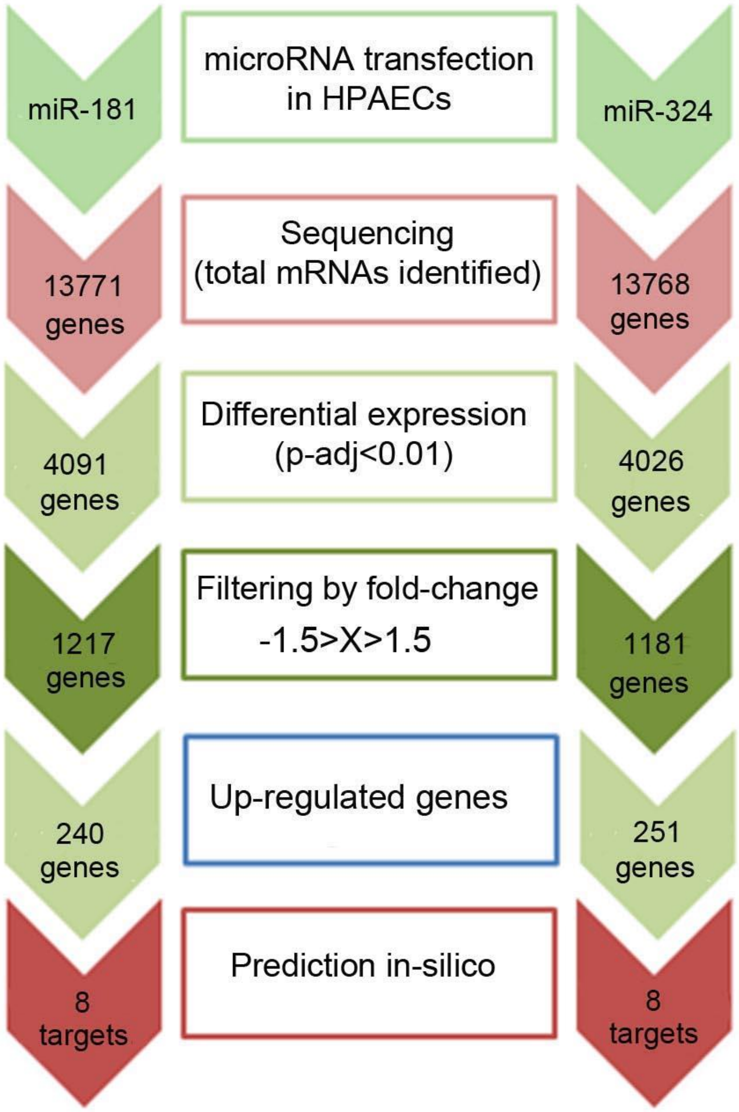

Supplementary Figure 15. Identification of predicted and validated gene targets upregulated by miR-181 and miR-324 in HPAECs. HPAECs were transfected with miRNA mimics and subjected to RNA sequencing. List of transcripts with fold-change >1.5 and adjusted p value <0.01 that were up-regulated by miR-181 and 324 was compared with the list of predicted miRNA gene targets, providing 8 targets of miR-181 and 8 targets of miR-324 of potential interest. Target prediction was carried out with TargetScan Human, miRecords and Ingenuity Expert Findings (IPA).

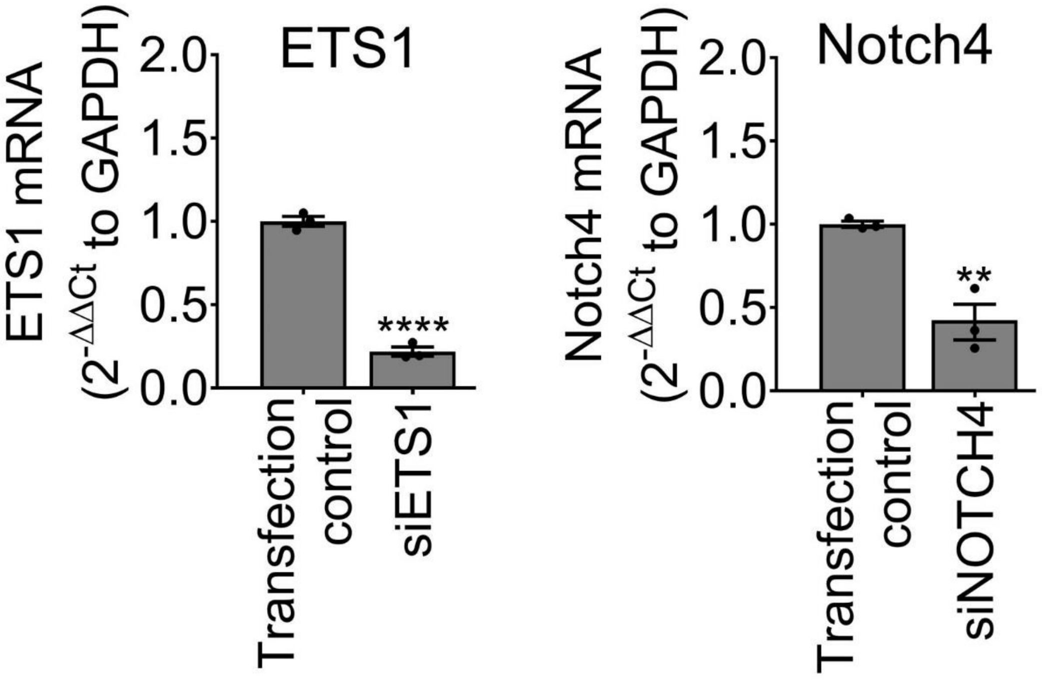

Supplementary Figure 16. **ETS-1 and Notch4 expression in siRNA-transfected HPAECs.** Cells were transfected with control siRNA (Transfection control), ETS-1siRNA or Notch4 siRNA, as indicated and changes in the mRNA expression of ETS-1 and Notch4 were studied 48h post-transfection. Bars are means ± s.e.m. **P<0.01, ****P<0.0001, comparison with transfection control, unpaired t-test, n=3. All n numbers represent independent experiments. Source data are provided in Supplemental Source Data file.

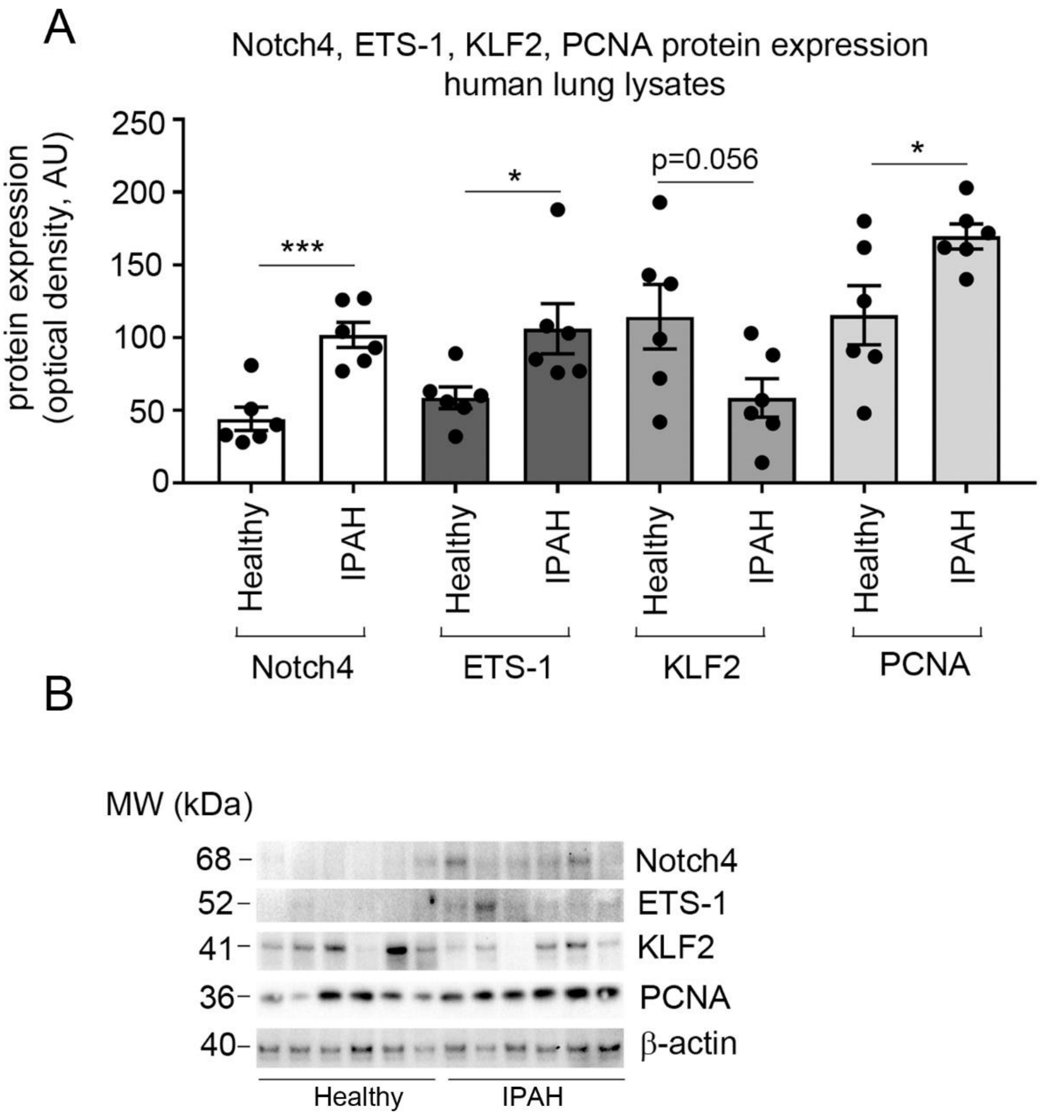

Supplementary Figure 17. Expression of Notch4, ETS-1, KLF2 and PCNA in lung lysates from healthy individuals and IPAH patients. (A) Graph and (B) western blots show expression of proteins of interest, as indicated. Mean optical density ± s.e.m. *P<0.05, ***P<0.001, or as indicated; comparison of IPAH with corresponding controls, unpaired t-test; n=6. All n numbers represent biologically independent samples. Source data are provided in Supplemental Source Data file.

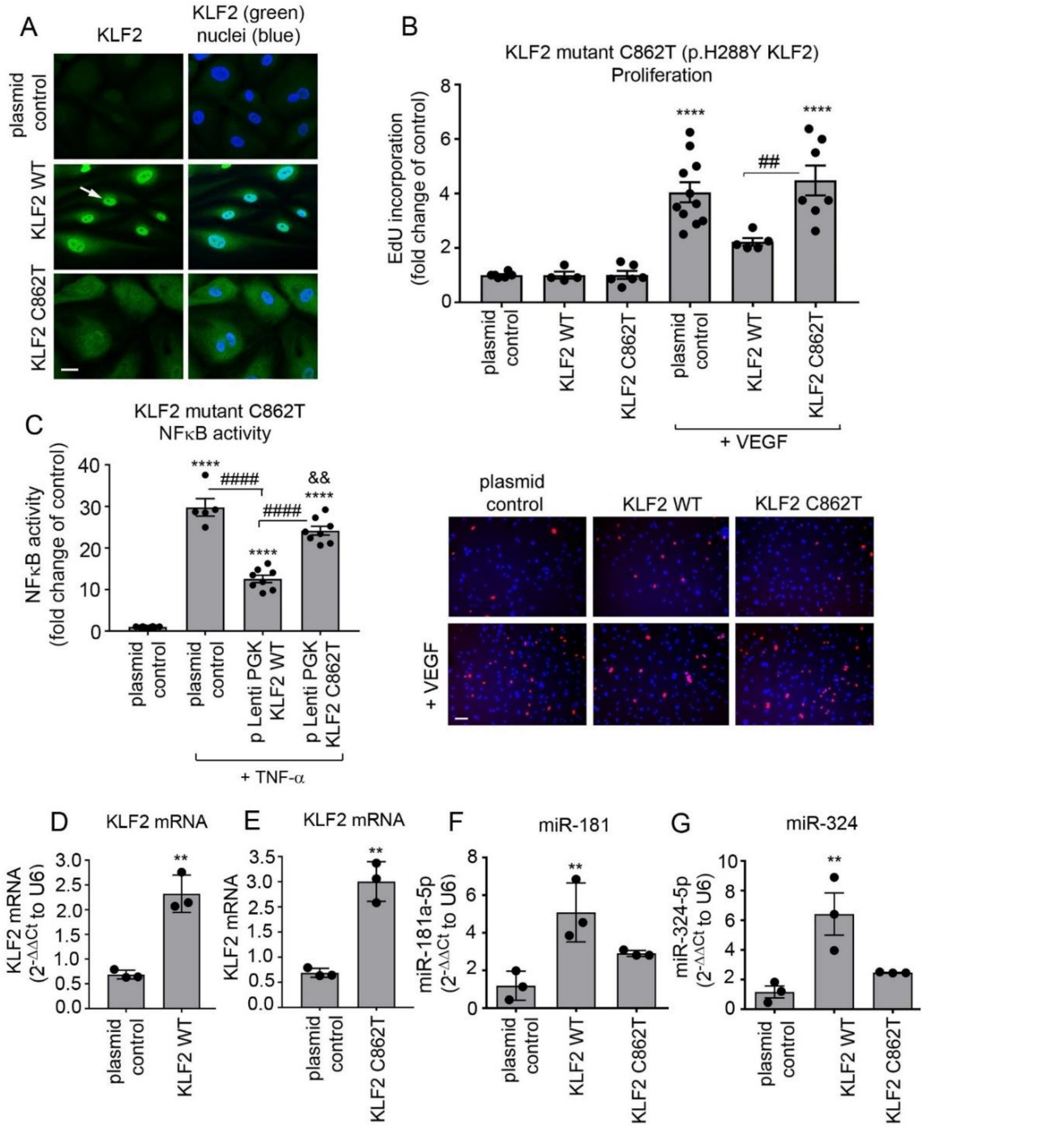

Supplementary Figure 18. KLF2 loss of function by p.H288Y mutation. HPAECs were transduced with KLF2 WT or KLF2 c.862C>T (C862T KLF2) lentiviral particles. (A) Reduced nuclear localization of KLF2 C862T in HPAECs. Arrow points to nuclear localization of wildtype (WT) KLF2; nuclei are blue and KLF2 is green; Bar=10 µm. No effect of C862T KLF2 on (B) VEGF-induced endothelial proliferation (graph and images below the graph), (C) TNF-α-induced NFκB activation. (D-G) show expression levels of KLF2 mRNA, miR-181 and miR-324 in cells overexpressing KLF2 WT or C862T KLF2, as indicated. In images below the graph (B) nuclei are blue and EdU-incorporating nuclei are red; Bar=50 µm**.** Bar graphs show mean fold-changes of plasmid controls; $^{**}P<0.01$, $^{****}P<0.0001$, comparison with plasmid controls; $^{\&\&}P<0.01$, comparison between plasmid control+TNF-α and KLF2 C862T+TNF-α; $^{\#\#}P<0.01$, $^{\#\#\#\#}P<0.0001$, comparisons, as indicated. (B, C, F, G) One-way ANOVA with Tukey post-test, n=5-8; (D, E) unpaired t-test, n=3. All n numbers represent independent experiments. Source data are provided in Supplemental Source Data file.

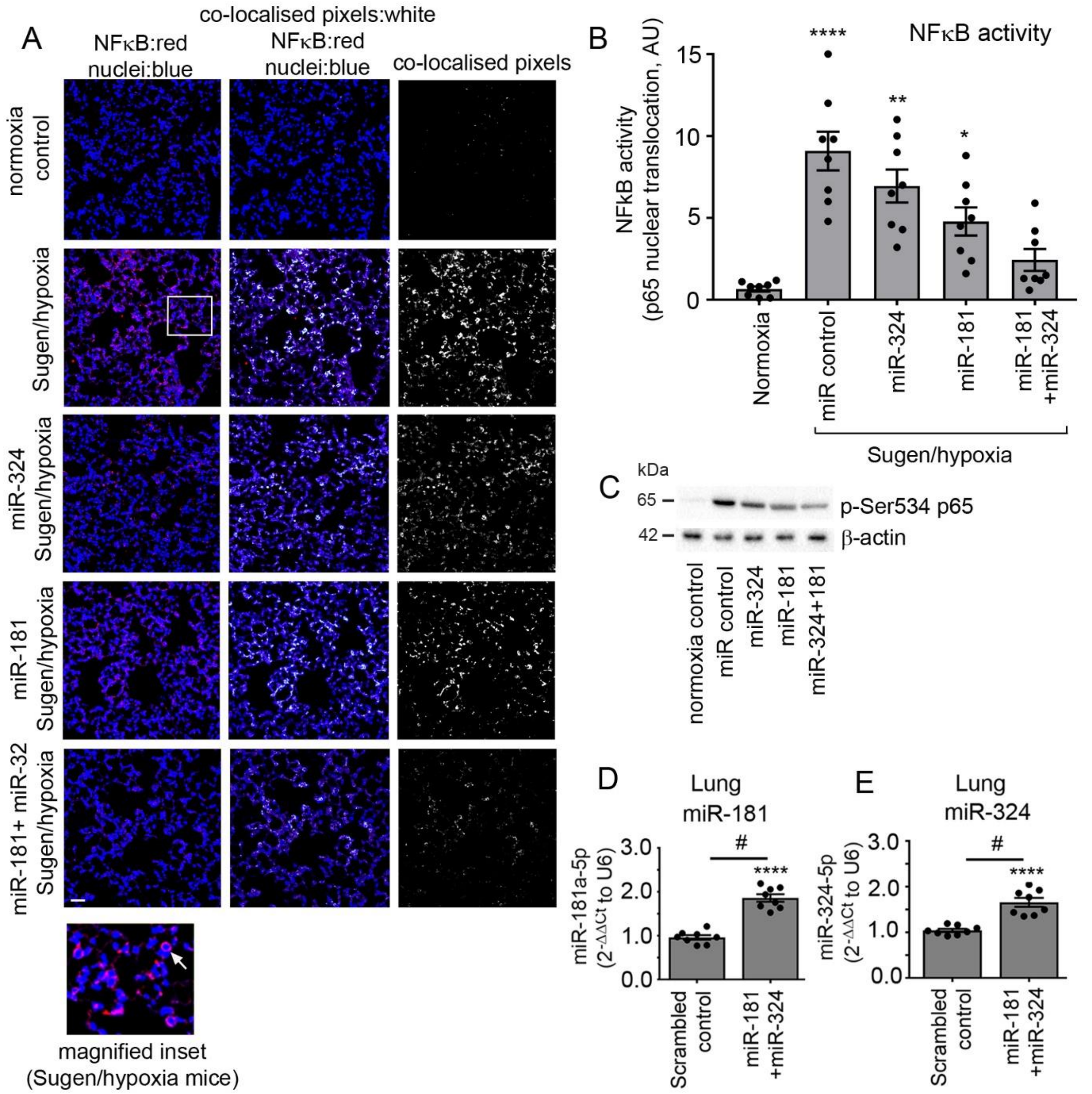

Supplementary Figure 19. NFκB activity in the lungs of Sugen/hypoxia mice treated with control miRNA, miR-181 and miR-324. (A) Nuclear localization of p65NFκB in control or Sugen/hypoxia mice treated with miRNA control or with single or combined miR-181 and miR-324, two days after post-injection. Nuclear localization of p65 NFκB was studied by immunofluorescence and confocal microscopy. In confocal images on the left, nuclei are blue (DAPI) and p65NFκB is red. White pixels in the middle and the right images show colocalization of p65NFκB with nuclear DNA (Image J). In the magnified inset below the panel, the arrow points to nucler localization of p65 NFκB. Bar=25μm. (B) NFκB activity in the lungs of mice treated, as indicated; n=8. (C) Representative western blots show changes in the phosphorylation of p65 NFκB on Ser534 in mouse lungs; (D, E) miR-181 and miR-324 levels in the lungs of Sugen/hypoxia mice following treatment with scrambled miRNA control

or miR-181 and miR-324. *P<0.05, **P<0.01, ****P<0.0001; comparison with controls; #P<0.05, comparison, as indicated. One-way ANOVA with Tukey post-test (B) or unpaired t-test (D, E), as appropriate; n=8. All n numbers represent biologically independent samples. Source data are provided in Supplemental Source Data file.

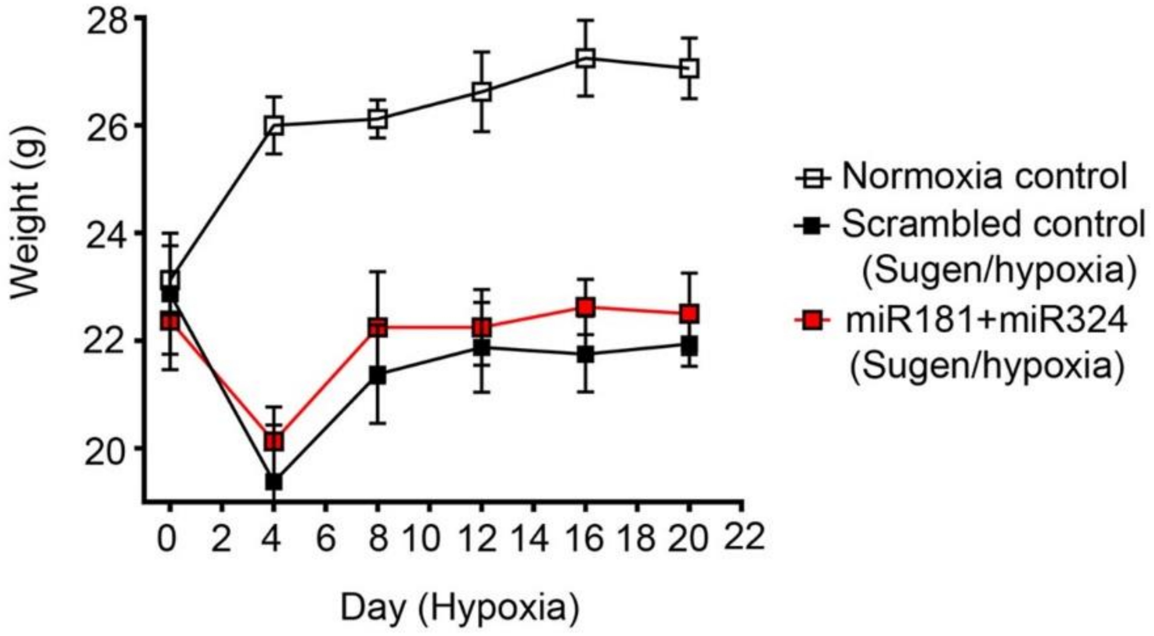

Supplementary Figure 20. Weight migration in mice from different treatment groups. n=8. All n numbers represent biologically independent samples. Source data are provided in Supplemental Source Data file.

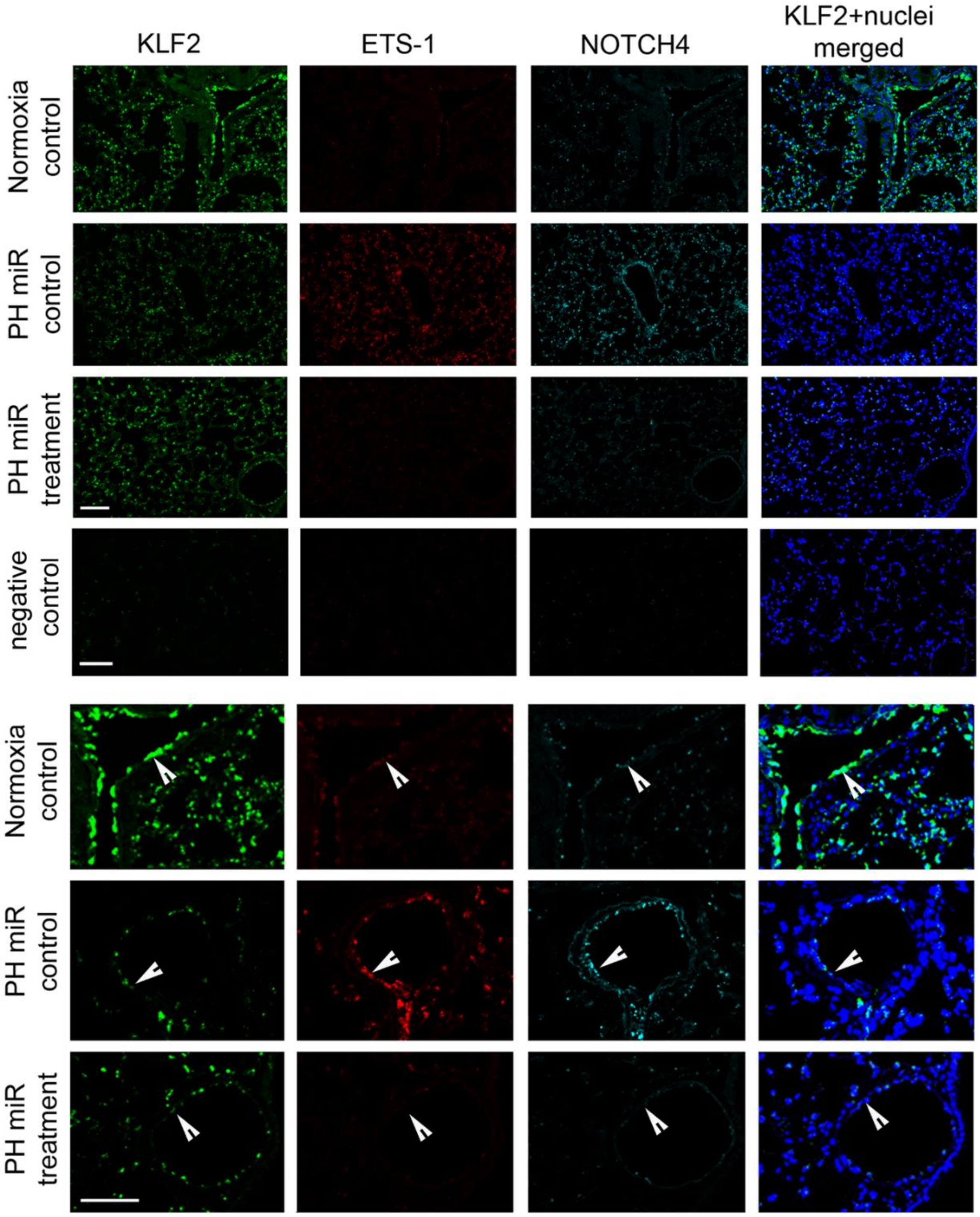

Supplementary Figure 21. Localization of KLF2, ETS-1 and NOTCH4 mRNA in the lungs of control and Sugen/hypoxia mice. Localization of specific transcripts was carried out with fluorescent RNAscope *in situ* hybridization followed by fluorescent confocal microscopy analysis of lung tissues from control, pulmonary hypertensive and miR-181- and miR-324-treated Sugen/hypoxia mice. Bottom panel shows magnified images of lung tissues. Each fluorescent dot corresponds to a single transcript; arrowheads point to endothelial cells. Bar=25µm; n=8.

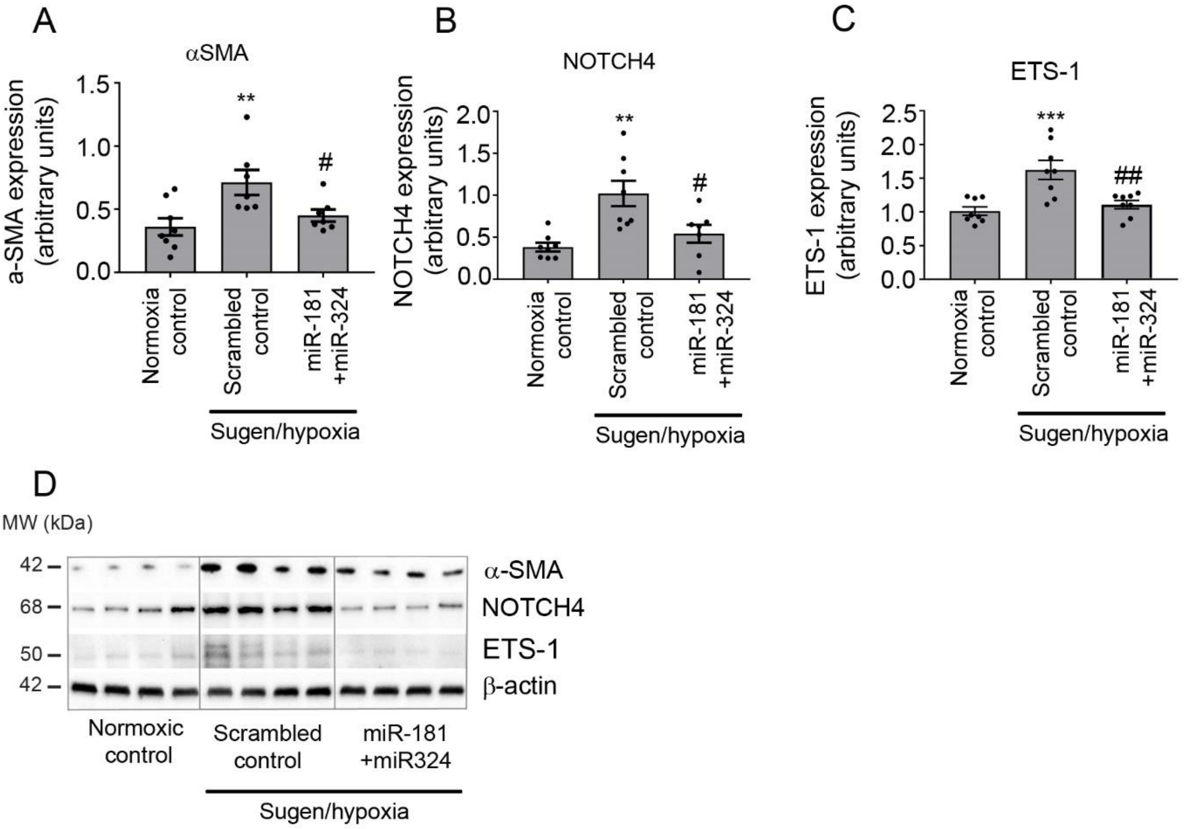

Supplementary Figure 22. **Changes in selected targets of miR-181 and miR-324 in the lungs of Sugen/hypoxia mice.** Protein levels of (A) α-SMA; (B) NOTCH4 and (C) ETS-1 in the lungs of normoxic control mice, Sugen/hypoxia mice injected with control miRNA or treated with miR-181 and miR-324 mimics, as indicated. (D) Representative western blots corresponding to graphs (A-C). Bars are means± s.e.m., one-way ANOVA with Tukey post-test. **P<0.01, ***P<0.001, comparison with normoxia control; $^{\#}$P<0.05, $^{\#\#}$P<0.01 comparisons with Sugen/hypoxia scrambled control; n=8. All n numbers represent biologically independent samples. Source data are provided in Supplemental Source Data file.

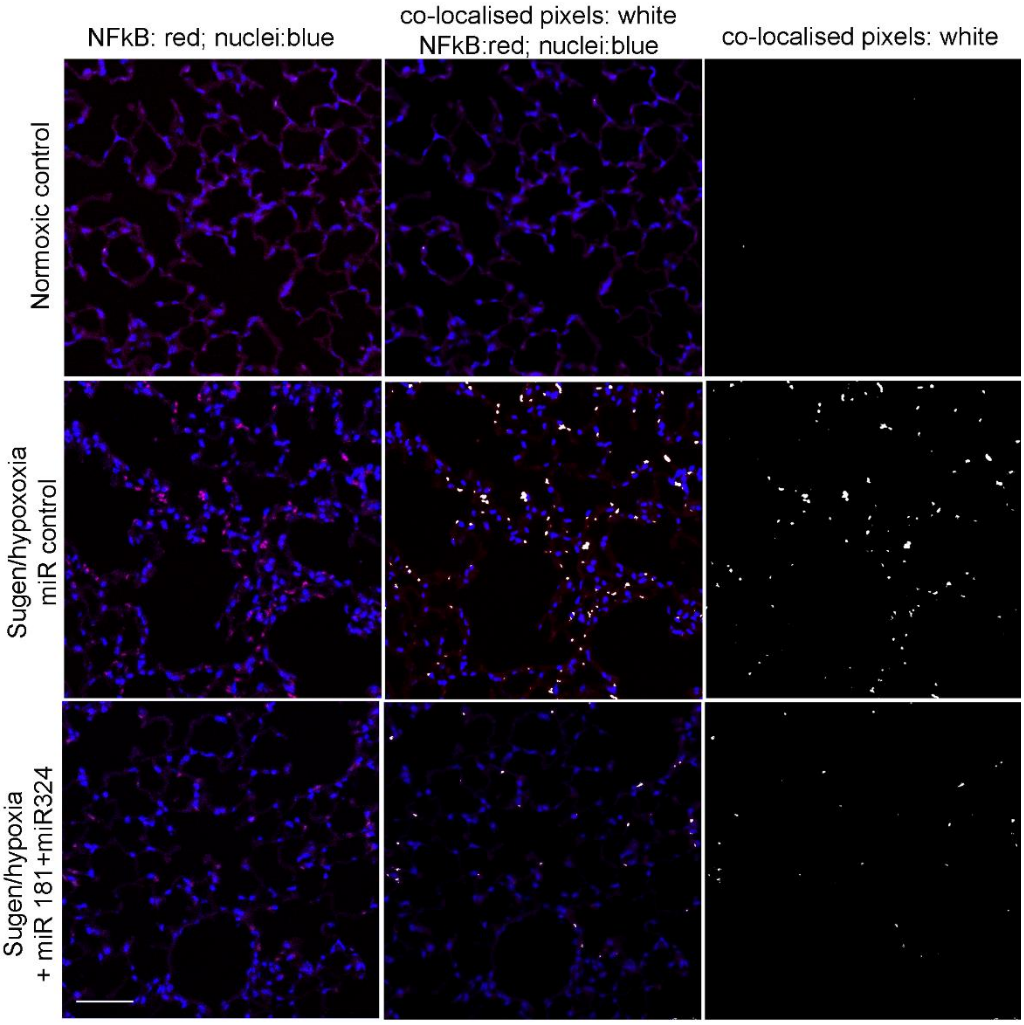

Supplementary Figure 23. Nuclear translocation of NFκB in the lungs of Sugen/hypoxia mice treated with control miRNA, miR-181 and miR-324. Nuclear localization of p65 NFκB was studied by immunofluorescence and confocal microscopy in Sugen/hypoxia mice treated by intravenous injection of control miRNA or combination of miR-181 and miR-324 for 21 days. In confocal images on the left, nuclei are blue (DAPI) and p65NFκB is red. White pixels in the middle and in the right images show colocalization of p65NFκB with nuclear DNA (Image J). Bar=50µm; n=8.

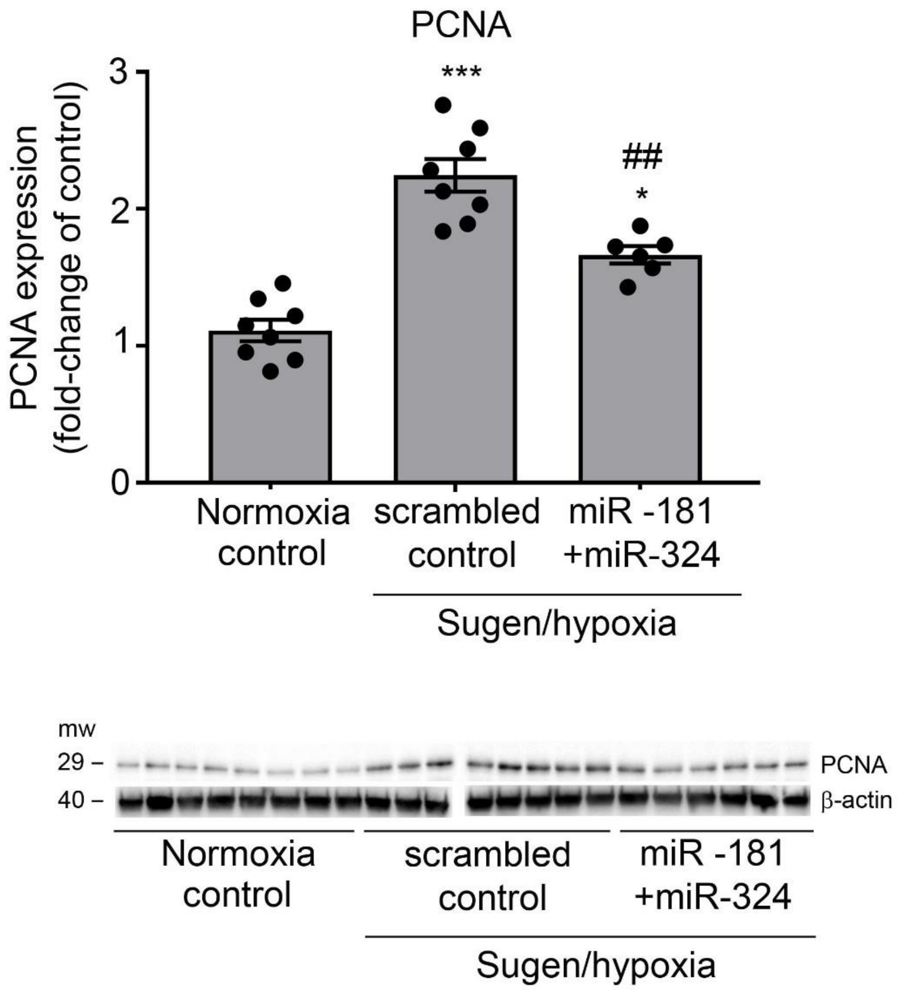

Supplementary Figure 24. Expression of proliferating cell nuclear antigen (PCNA) in the lungs of normoxia and Sugen/hypoxia mice treated, as indicated. *P<0.05, ***P<0.001, comparison with normoxia controls, $^{\#\#}$P<0.01, comparison with Sugen/hypoxia scrambled controls. One-way ANOVA, Tukey post-test; n=6-8. All n numbers represent biologically independent samples. Source data are provided in Source Data file.
